# Importin β1 Mediates Nuclear Entry of EIN2C to Confer the Phloem-Based Defense against Aphids

**DOI:** 10.3390/ijms24108545

**Published:** 2023-05-10

**Authors:** Kai Lu, Liyuan Zhang, Lina Qin, Xiaochen Chen, Xiaobing Wang, Meixiang Zhang, Hansong Dong

**Affiliations:** 1 State Key Laboratory of Crop Biology, College of Plant Protection, Shandong Agricultural University, Taian 271018, China; 2 Institute of Tropical Crop Genetic Resources, Chinese Academy of Tropical Agricultural Sciences, Danzhou 571737, China; 3 College of Life Sciences, Shaanxi Normal University, Xi’an 710019, China

**Keywords:** arabidopsis, aphids, EIN2 C-terminal portion (EIN2C), importin β1 (IMPβ1), nuclear import, phloem-based defense (PBD)

## Abstract

Ethylene Insensitive 2 (EIN2) is an integral membrane protein that regulates ethylene signaling towards plant development and immunity by release of its carboxy-terminal functional portion (EIN2C) into the nucleus. The present study elucidates that the nuclear trafficking of EIN2C is induced by importin β1, which triggers the phloem-based defense (PBD) against aphid infestations in Arabidopsis. In plants, IMPβ1 interacts with EIN2C to facilitate EIN2C trafficking into the nucleus, either by ethylene treatment or by green peach aphid infestation, to confer EIN2-dependent PBD responses, which, in turn, impede the phloem-feeding activity and massive infestation by the aphid. In Arabidopsis, moreover, constitutively expressed EIN2C can complement the *impβ1* mutant regarding EIN2C localization to the plant nucleus and the subsequent PBD development in the concomitant presence of IMPβ1 and ethylene. As a result, the phloem-feeding activity and massive infestation by green peach aphid were highly inhibited, indicating the potential value of EIN2C in protecting plants from insect attacks.

## 1. Introduction

The gaseous phytohormone ethylene (C_2_H_4_) regulates plant growth, development, and immunity via a signaling (signal transduction) pathway that uses EIN2 as the central regulator [1,2,3,4,5,6,7,8]. EIN2 is a 141-kD integral membrane protein of 1294 amino acids with structural features suited for ethylene signaling [1]. The N-terminal part of 461 amino acids forms 12 transmembrane domains [1], which enable EIN2 to partake in ethylene signal transduction from the endoplasmic reticulum (ER) membrane towards an intracellular physiological pathway [3]. At the opposite end, the longer C-terminal portion of 833 amino acids [1] contains a putative nuclear localization signal comprising 1262–1269 amino acids, which targets EIN2 or its C-terminal portion into the nucleus [9]. Following translocation, the C-terminal region of EIN2 is sufficient to activate ethylene responses associated with plant growth, development, and immunity [1,7,9,10,11]. Such a functional C-terminal fragment truncated from the EIN2 sequence was designated as EIN2 CEND [1,12] and is called EIN2C for the sake of convenience hereafter.

In response to the ethylene signal, EIN2 is proteolytically cleaved at its C-terminal region to produce length-varied EIN2C species. Many EIN2Cs have been confirmed to be the products of EIN2 protease cleavage [1,7,9,12,13,14]. The first characterized EIN2C, EIN2^454–1294^, was produced by polymerase chain reaction (PCR) amplification of EIN2 C-terminal partial sequence [1]. EIN2Cs have been shown to function in ethylene signaling [7,9,12,13,14,15,16]. After being expressed de novo in plants, particularly in Arabidopsis and tobacco, these EIN2Cs function to confer ethylene responses that associate with plant growth and immunity [1,9,12,14,15].

EIN2 is involved in plant immunity [1,17,18,19,20], including the Phloem-Based Defense (PBD) mechanism [21,22]. The PBD can effectively counterattack infestations of phloem-feeding insects, which are also called sap-sucking insects and, typically, include aphids [23]. The insects of this category are highly specialized in feeding from the phloem through stylet penetration of plant cells, which presents a unique stress on plant fitness [21,22,23,24,25,26]. In response, the PBD is established by massive production of β-1,3-glucan callose and phloem proteins Phloem Protein 2-like A1 (PP2-A1) and Phloem Protein 2-like A1 (PP2-A2) in sieve tubes [21,27]. Callose coagulation on sieve plates and phloem phase plugging and callose closure of sieve pores provide a strong physical barrier that effectively impedes the phloem-feeding activities of the insects, thereby blocking continued infestation in the plant [10,28,29]. In plants under aphid attack, ethylene signaling is critical for the PBD establishment usually in leaves via deposition of callose, which is produced by the Glucan Synthesis-like (GSL) enzymes [21,22,28], and accumulation of lectin-type phloem proteins [11,22,25]. Lectin-type phloem proteins that have been characterized as essential components of the PBD include PP2-A1 and PP2-A2 in Arabidopsis [11,25]. In essence, both GSL and phloem proteins are produced following expression of the cognate genes *GSL5*, *PP2-A1*, and *PP2-A2* [11,28]. These genes are regarded as critical PBD response genes, which are expressed upon induction either by plant treatment with ethylene or by plant colonization with aphids, and which are expressed in an EIN2-dependent manner [10,11,21,22,28,30].

EIN2 becomes activated upon ethylene perception by any of the five functionally redundant receptors [4,31,32]. Once ethylene is sensed by an ethylene receptor, EIN2 is dephosphorylated to liberate EIN2Cs [12]. Then, some EIN2Cs move into the nucleus [12] to activate a transcriptional cascade that confers plant immunity and ethylene responses [11,33,34]. EIN2C translocation from the ER into the nucleus is a pivotal step towards the transcriptional cascade. In eukaryotic cells, the import of macromolecules, which are referred to as cargoes and mainly include proteins and RNA, into the nucleus most often requires nuclear import carrier proteins called importins (IMP). IMPs are presented by α and β forms and mediate cargo trafficking most frequently in a complex of either “cargo–IMPβ” or “cargo–IMPα–IMPβ”, wherein the IMPα is a substrate adaptor, or, rarely, in a form of “cargo–IMPα”, wherein the IMPα directly serves as a transporter [35,36,37]. Arabidopsis genome encodes 8 IMPα and 18 IMPβ proteins [38], but none was associated with the nuclear import of EIN2Cs.

This gap has been bridged by the present study demonstrating the IMP loss-of-function imp Arabidopsis mutants with respect to molecular trafficking engaged in phytohormone-mediated basal defense pathways in the plant. By investigating the WT plant, *imp* mutants including those defected in IMPβ1 (synonym KPNB1), and *impβ1*-complemented (*impβ1/IMPβ1-RFP*) transgenic lines, IMPβ1was characterized as an efficient facilitator for the nuclear import of EIN2^454–1294^, which is the first identified EIN2C [1]. The present study was devised to elucidate if IMPβ1-mediated nuclear import of EIN2C also regulates plant PBD against aphid infestations. IMPβ1 and EIN2C were determined to inhibit the phloem-feeding activity of green peach aphid (*Myzus persicae* Sulzer), a model species of insects attacking Arabidopsis [10,11,25]. Evidence detailed below will demonstrate that IMPβ1 mediates the nuclear transport of EIN2C to activate PBD responses against aphid infestations in Arabidopsis.

## 2. Results

### 2.1. IMPβ1 Strongly Affects Arabidopsis Resistance to Green Peach Aphid

**Figure 1:**
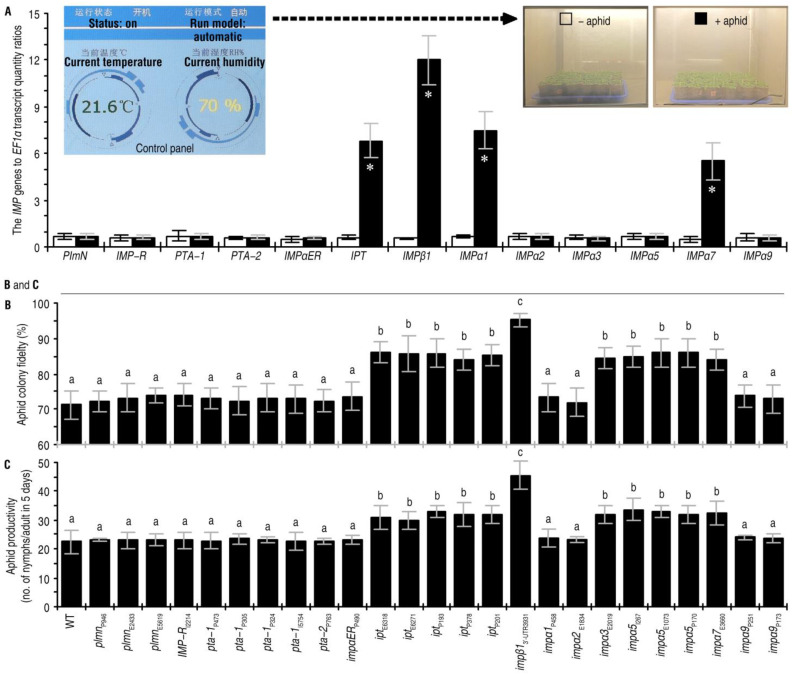
Five IMPs, including IMPβ1, are related to Arabidopsis response to green peach aphid infestations. (**A**) Differential expression of 13 *IMP* genes in Arabidopsis plants in the presence and absence of aphid infestations. Leaves of 20-day-old plants were artificially colonized with 10-day-old nymphs of green peach aphid (+aphid), while plants in the control group remained free from aphids (−aphid). Two days later, RNA was isolated from leaves and analyzed by qRT-PCR using *EF1α* as a reference gene. Data shown are mean values ± standard deviation (SD) estimates of results from six independent experiments, each involving 15 plants. Asterisks indicate significant differences between plants with and without aphid infestations. (*n* = 6, * *p* < 0.001; Student’s *t*-test.) (**B**,**C**) Aphid colony fidelity in two days and productivity rates in five days after placement on leaves of the tested genotypes (listed at **bottom**). Data shown are mean values ± SD estimates of results obtained from six independent experiments, each involving 200 aphids colonized on 10 plants. The different letters on the graphs indicate significant differences, as assessed using Duncan’s new multiple-range test (*p* < 0.05).

This study began with investigating 13 Arabidopsis *IMP* genes (Figure 1A), that have loss-of-function mutants generated previously by T-DNA insertion (Supplementary Table S1). To look for functional links between the IMPs and Arabidopsis defenses against green peach aphid, qRT-PCR was performed to quantify *IMP* transcript levels in plants of the Arabidopsis ecotype Col-3 during the aphid infestation. Expression of *IPT*, *IMPβ1*, *IMPα1*, and *IMPα7* were significantly increased by artificial colonization of the plants with aphids (Figure 1A). Quantities of *IPT*, *IMPβ1*, *IMPα1*, and *IMPα7* transcripts in aphid-colonized plants were 5.8-, 11.0-, 6.6-, and 4.7-times higher than those in aphid-free control plants, respectively. In contrast, the other nine *IMP* genes did not show significant transcriptional changes in responses to aphid colonization (Figure 1A).

To determine if the *IMP* genes affect Arabidopsis defenses, colony fidelities and reproduction rates of aphids placed on leaves of the WT and *imp* plants were conveyed. If the aphid colony fidelity was higher on an *imp* mutant compared with the WT plant, the *IMP* was thought to be a positive regulator of Arabidopsis resistance to aphid colonization. The aphid colony fidelity rates on the *ipt*, *impβ1*, *impα3*, *impα5*, and *impα7* mutants were significantly increased compared with the WT plants. Thus, IPT, IMPβ1, IMPα3, IMPα5, and IMPα7 contributed substantially to plant resistance (Figure 1B). Meanwhile, an IMP was thought inhibitive to aphid reproduction if the reproduction rate was greater on the *imp* mutant than on the WT plant. The *imp* mutants that favored the colonization of aphid (Figure 1B) also supported aphid reproduction (Figure 1C). The highest rate of aphid reproduction occurred on the *impβ1* mutant (Figure 1C). These data suggested that IPT, IMPβ1, IMPα3, IMPα5, and IMPα7 contribute to Arabidopsis resistance against green peach aphid infestation and that IMPβ1 is the most influential resistance constituent.

### 2.2. IMPβ1 Supports PBD Defense Gene Expression but Does Not Affect Bacterial Infection

**Figure 2:**
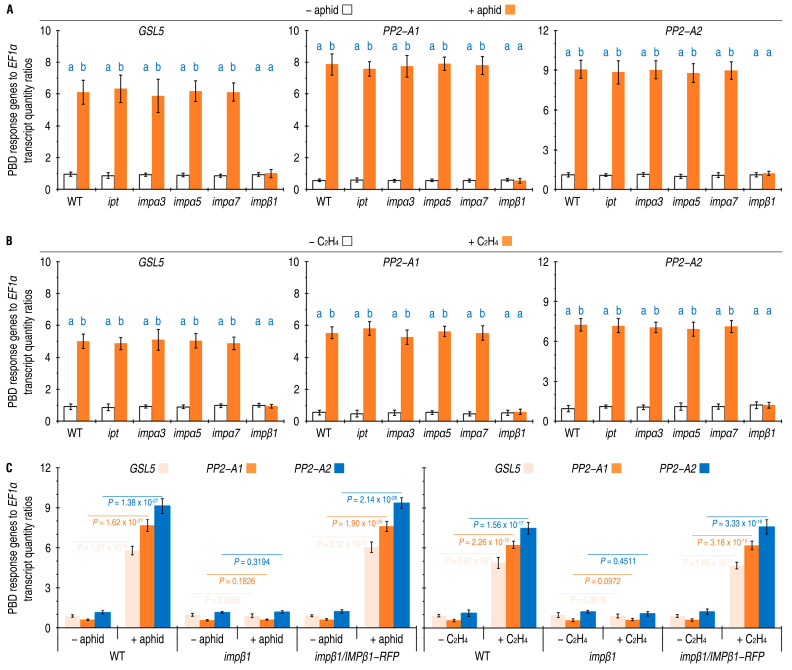
The five IMPs, including IMPβ1, affect PBD response gene expression in Arabidopsis plants responding to aphid infestation or ethylene (C_2_H_4_) treatment. (**A**) The gene expression in leaves of the WT and *imp* mutant plants that remained free from or artificially colonized with aphids. (**B**) The gene expression in leaves of the WT and *imp* mutant plants that were incubated in air or in 10 µL/L ethylene. (**C**) The gene expression in leaves of the WT, *impβ1* mutant, and *impβ1*-complemented (*impβ1/IMPβ1-RFP*) transgenic plants that remained free from or artificially colonized with aphids. In (**A**,**B**), 20-day-old plants were treated differently as designed, and, 24 h later, RNA was isolated from the treated or equivalent leaves and analyzed by qRT-PCR, which used *EF1α* as a reference gene. Data shown are mean values ± SD estimates of results obtained from six independent experiments, each involving 200 aphids colonized on 10 plants. In (**A**,**B**) The different letters on the graphs indicate significant differences, as assessed using Duncan’s new multiple-range test (*p* < 0.01). In (**C**), *p*-values obtained in the two-sided student’s *t*-test are provided.

Defense response genes *PP2-A1*, *PP2-A2*, and *GSL5* have been shown to be essential constituents of the PBD [10,11,21,22,25,28]. To determine whether IPT, IMPβ1, IMPα3, IMPα5, or IMPα7 affects Arabidopsis PBD, we conducted qRT-PCR analysis on the leaves of both WT and *imp* mutant plants. These plants were either free from aphids or artificially colonized with 10-day-old nymphs of green peach aphids. After 24 h, we compared the expression levels of *PP2-A1*, *PP2-A2*, and *GSL5* in the leaves. The qRT-PCR data indicated that *PP2-A1*, *PP2-A2*, and *GSL5* were expressed at the steady-state levels in leaves of all plants without aphid colonization, but these genes considerably increased their transcript quantities after aphid colonization in the WT plant (Figure 2A). Aphid-induced enhancements of *PP2-A1*, *PP2-A2*, and *GSL5* expression were also found in the *ipt*, *impα3*, *impα5*, and *impα7* mutants (Figure 2A). However, colonization by aphid did not have substantial effects on expression levels of *PP2-A1*, *PP2-A2*, and *GSL5* in the *impβ1* mutant, and did not cause significant changes in transcript amounts of the three genes from the steady-state levels (Figure 2A).

Similar results were obtained from plants incubated in air and in 10 µL/L ethylene. The *ipt*, *impα3*, *impα5*, and *impα7* mutants resembled the WT plants in response to the externally applied ethylene, which highly enhanced expression of *PP2-A1*, *PP2-A2*, and *GSL5* in leaves of these plants (Figure 2B). In 24 h after ethylene treatment, expression levels of *GSL5*, *PP2-A1*, and *PP2-A2* were increased accordingly by five, nine, and six times on average in comparison to the ready-state expression extents. On the contrary, the *impβ1* mutant failed to display ethylene-enhanced expression of the PBD response genes. Instead, the expression of *PP2-A1*, *PP2-A2*, and *GSL5* in leaves of ethylene-treated *impβ1* plants remained around the steady-state levels as found in the absence of ethylene treatment (Figure 2B).

The effects of *IMPβ1* on *PP2-A1*, *PP2-A2*, and *GSL5* expression were confirmed by investigating *impβ1*-complemented (*impβ1/IMPβ1-RFP*) transgenic Arabidopsis lines. The *impβ1/IMPβ1-RFP* lines were generated by transformation of the *impβ1* mutant blossoms with the plant binary vector pCAMB1301 [10] that was constructed to carry a recombinant of the native *IMPβ1* promoter (*IMPβ1P*), *IMPβ1* coding sequence (*IMPβ1*), and *red-fluorescent protein* (*RFP*) gene (Supplementary Figure S1A). Well-characterized four *impβ1/IMPβ1-RFP* lines (#1 to #4) shared common characters, resembling the WT plant in growth and development (Supplementary Figure S1B). Here, *impβ1/IMPβ1-RFP* #1 (simply called *impβ1/IMPβ1* hereafter) was compared with the mutant and WT plants in terms of *PP2-A1*, *PP2-A2*, and *GSL5* expression after plant colonization by aphids or ethylene treatment. Based on qRT-PCR analyses carried out 24 h later, quantities of *PP2-A1*, *PP2-A2*, and *GSL5* transcripts detected in leaves were significantly increased by aphid colonization or ethylene treatment in *impβ1/IMPβ1-RFP* plants, as well as in WT plants (Figure 2C). In both the WT and *impβ1/IMPβ1-RFP* plants, expression levels of *GSL5*, *PP2-A1*, and *PP2-A2* gained approximately six-, twelve-, and seven-fold increases by aphid colonization, and about five, nine, and six times by ethylene treatment, respectively. In the *impβ1* mutant, however, neither aphid colonization nor ethylene treatment provided evident noticeable in *GSL5*, *PP2-A1*, and *PP2-A2* expression, which instead remained around the steady-state levels (Figure 2C).

Taken together, these analyses suggest that *IMPβ1* is essential, but *IPT*, *IMPα3*, *IMPα5*, and *IMPα7* are not, for the PBD response gene expression induced either by aphid colonization or by ethylene treatment in Arabidopsis. In essence, IMPβ1 is likely to partake in ethylene signaling for the PBD regulation. Thus, IMPβ1 is focused in further studies stated hereafter.

We have confirmed that IMPβ1 supports the resistance to aphids in Arabidopsis, and we are interested in exploring whether IMPβ1 affects the resistance to pathogens. To our surprise, IMPβ1 did not affect the plant defense against the bacterial pathogen *Pseudomonas syringae* pv. *tomato* (*Pst*). The WT, *impβ1*, and *impβ1/IMPβ1-RFP* plants displayed similar sensitivities to *Pst*, allowing it to vigorously propagate in leaf tissues and finally cause disease with visible symptoms (Figure S2A,B). IMPβ1 was also unrelated to the plant resistance against *Pectobaterium carotovora* subsp. *carotovora* (*Pcc*), the bacterial pathogen that causes soft rot in cruciferous plants. *Pcc* causes necrosis in Arabidopsis leaves after spray inoculation in the absence of water films. Indeed, *Pcc* bacteria propagated in leaf tissues and caused severe necrosis symptoms irrespectively of the plant genotypes (Supplementary Figure S2C,D). Therefore, IMPβ1 has no visible function in the plant resistance against these virulent bacterial pathogens.

### 2.3. IMPβ1 Directly Interacts with EIN2C in Plant Nuclei

**Figure 3:**
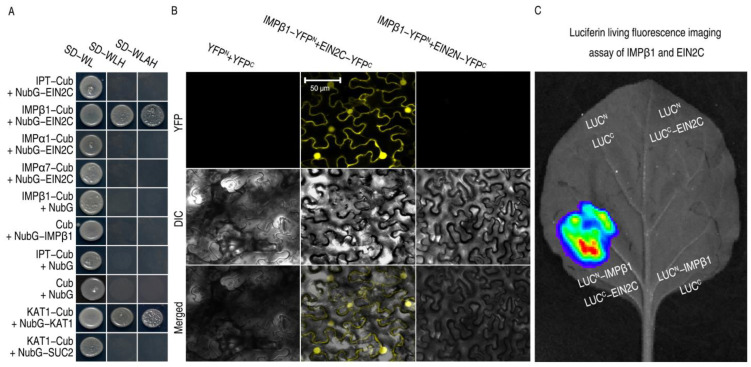
IMPβ1 directly interacts with EIN2C in plant nuclei. (**A**) SUB-Y2H assays of EIN2C and the IMPs. Multiple positive and negative controls are partly shown here. The positive control was provided by combination of the potassium ion (K^+^) channel protein KAT1 (K^+^ Arabidopsis thaliana 1). One of the negative controls was achieved using SUC2 (sucrose transport protein 2). Results shown represent three independent experiments. (**B**) The YFP BiFC of IMPβ1 and Ein2C expressed in *Nicotiana benthamiana* leaves was observed using confocal laser-scanning microscope (CLSM). (**C**) Luciferin (Luc) living fluorescence imaging (LLFI) assays performed on *N. benthamiana* leaves transformed with IMPβ1 and Ein2C proteins. Luc^N^ and Luc^C^ represent the N-terminal and C-terminal halves of the Luc fusion constructs of the tested proteins. The leaf image represents six leaves from two plants tested in three experimental replicates.

Our study found that IMPβ1 may be involved in the regulation of PBD by ethylene signaling. EIN2 is a critical factor in the positive regulation of the ethylene signaling pathway. To infer a functional relationship between EIN2 and IMPβ1, the IMPβ1 was subjected to protein–protein interaction assays with EIN2C (EIN2^454–1294^) using a split-ubiquitin-based yeast-two hybrid (SUB-Y2H) system. An interaction occurred specifically between EIN2C and IMPβ1, but not between EIN2C and any of the other IMPs (*IPT*, *IMPα3*, *IMPα5*, and IMPα7) (Figure 3A). The specificity was further evidenced by the positive control using KAT1 and the negative control using SUC2 (Figure 3A). To confirm and locate the IMPβ1 and EIN2C interaction, we carried out bimolecular fluorescence complementation (BiFC) assays. IMPβ1 was fused to the N-terminal half of YFP (YFP^N^), generating the IMPβ1:YFP^N^ fusion protein, while EIN2N (EIN2N^1−453^) and EIN2C were fused to the YFP C-terminal half (YFP^C^), forming the EIN2N:YFP^C^ and EIN2C:YFP^C^ fusion protein (Figure 3B). An interaction was observed only between IMPβ1:YFP^N^ and EIN2C:YFP^C^ but not EIN2N:YFP^C^, and the interaction was found in the nucleus (Figure 3B and Figure S3). The IMPβ1-EIN2C interaction was corroborated by the luciferase assay performed on leaves of tobacco leaves (Figure 3C). Clearly, IMPβ1 interaction with EIN2C, creating a molecular basis for the possibility that IMPβ1 mediates EIN2C trafficking into the nucleus.

### 2.4. IMPβ1 Targets EIN2C into Plant Nuclei in Response to Ethylene

Subcellular localization of IMPβ1 protein is affected by ethylene. In the assay, the *impβ1/IMPβ1-RFP* seedlings were growing continuously in air and were shifted into ethylene, respectively. Twelve h later, leaves were excised from the plants and stained with 4,6-diamidino-2-phenylindole (DAPI), a blue-fluorescent compound that is permeable to membranes, has a high affinity with DNA, and is widely used as a molecular marker of nuclei [39,40]. CLSM clearly visualized the IMPβ1:RFP fusion protein presented in leaf epidermal cells (Supplementary Figure S4A). In those cells, IMPβ1:RFP was found in cytoplasmic and nuclei, before plant treatment with ethylene. After ethylene was applied to the plants, IMPβ1:RFP decreased its localization in the plasma membrane and cytoplasm and more co-localized with DAPI on the nucleus (Supplementary Figure S4A).

Ethylene enhanced the nuclear localization of IMPβ1 protein, which was further confirmed by transient expression assays. In the assay, leaves of WT tobacco plants were transformed with the *35S:IMPβ1:YFP* construct. These plants were incubated in air and ethylene, respectively. Leaves of these transformed plants were stained with DAPI, and observed by CLSM. In CLSM imaging, the IMPβ1:YFP fusion protein was localized in cell membranes and nuclei, in the leaves of transformed plants growing in air (Supplementary Figure S4B). In the presence of ethylene, IMPβ1:YFP fusion protein was abundantly expressed, and co-localized with DAPI were colocalized to nuclei in the leaves of transformed plants (Supplementary Figure S4B). Clearly, ethylene facilitates the localization of IMPβ1 in plant nucleus.

**Figure 4:**
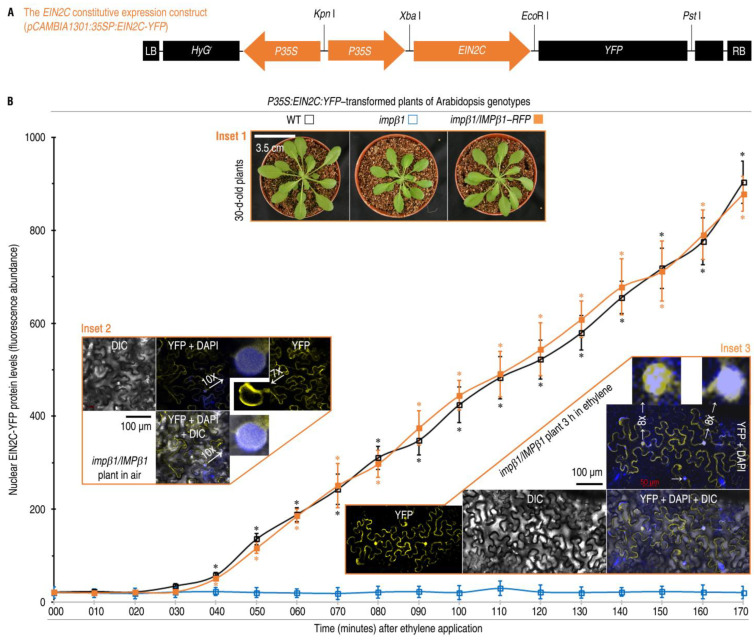
The EIN2C-YFP fusion protein moves into nuclei of Arabidopsis plants in the concomitant presence of IMPβ1 and ethylene. (**A**) Diagram of the fusion gene expression construct. (**B**) Chronological changes in relative concentrations of the fusion protein localized to plant nuclei. Leaves of the indicated plants (inset 1) were transformed with the construct shown in (**A**). These plants were shifted into 10 µL/L ethylene to grow for 40 h. Excised leaves were stained with DAPI and observed 10 min later by CLSM. Blue, orange, and black curves indicate the fluorescence abundance of EIN2C-YFP in the nucleus in WT, *impβ1,* and *impβ1/IMPβ1-RFP* plants, respectively. Each CLSM image (insets 2 and 3) represents six leaves from three plants. The quantitative data are shown as mean values ± SD estimates (*n* = six leaves). Asterisks indicate significant differences in WT, *impβ1/IMPβ1-RFP* compared with *impβ1* plants (* *p* < 0.05; Student’s *t*-test).

Consistent with ethylene-facilitated nuclear localization of IMPβ1, EIN2C was found to move into the nucleus in the concomitant presence of ethylene and a functional *IMPβ1*. In the assay, *EIN2C-YFP* was linked to *P35S* (Figure 4A) and transferred into the WT, *impβ1*, and *impβ1*/*IMPβ1-RFP* plants (Figure 4B inset 1). These transformed plants were incubated in 10 µL/L ethylene for 40 h. In the subsequent 3 h, EIN2C was constitutively expressed to similar levels in all plants no matter if they were incubated in air or ethylene, but *IMPβ1* was expressed only in the WT and *impβ1*/*IMPβ1-RFP* plants incubated in ethylene (Supplementary Figure S5). In the WT and *impβ1*/*IMPβ1-RFP* plants, the EIN2C-YFP fusion protein started to move into nuclei after 40 min of incubation in ethylene and displayed sharp increases in amounts of the nuclear localization subsequently in 50–170 min (Figure 4B). Consistently, CLSM of the *impβ1*/*IMPβ1-RFP* leaves, the EIN2C-YFP protein was localized to the endoplasmic reticulum (ER) membrane and nuclear envelope, and a minor amount of this protein was found in the nucleus, which was clearly visualized by DAPI, when the plants were incubated in air (Figure 4B inset 2 and Supplementary Figure S6). On the contrary, EIN2C-YFP colocalized to nuclei with DAPI in the plants as observed at the 3rd h after ethylene application (Figure 4B inset 3 and Supplementary Figure S6). During the period of observation, the *impβ1* mutant did not support the nuclear trafficking of EIN2C-YFP in comparison to the background readings (Figure 4B blue curve). We examined the co-localization of Impβ1-RFP and EIN2C-YFP. In air, IMPβ1 and EIN2C were co-located in cell membrane. However, IMPβ1 and EIN2C were co-located in nuclei in plant leaves treated with ethylene (Supplementary Figure S7). Clearly, IMPβ1 targets EIN2C into the nucleus in response to the exogenous ethylene.

### 2.5. IMPβ1-Mediated Nuclear Import of EIN2C Confers PBD Defense Responses

**Figure 5:**
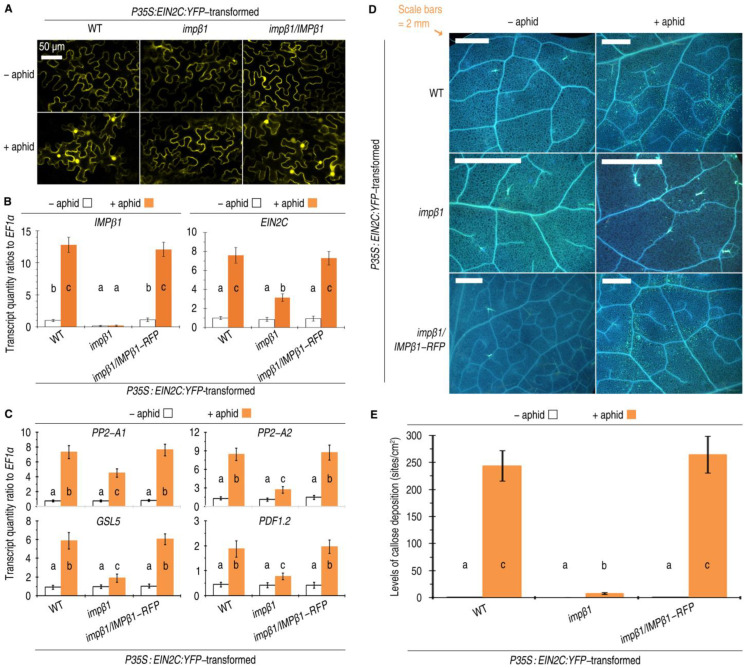
Overproduced EIN2C localizes to nuclei and confers PBD responses in Arabidopsis plants in the concomitant presence of *IMPβ1* and ethylene. (**A**) CLSM images of leaves from the indicated plants 60 h after transformation with the *P35S:EIN2C:YFP* construct and 24 h after artificial colonization with aphids. Each image represents 18 leaves from nine plants tested in three independent experiments. (**B**,**C**) Analyses by qRT-PCR to quantify the gene expression in leaves of the indicated plants 24 h after colonization with aphids or remained free from aphids. (**D**,**E**) Images showing callose deposition (blue dots) on leaves from plants 24 h after colonization with aphids or remained free from aphids. Each image represents 18 leaves randomly excised from 30 plants investigated in six independent experiments. Extents of callose deposition. (**B**,**C**,**E**) Data have been expressed as the mean ± SD (*n* = six); the different letters on the graphs indicate significant differences, as assessed using Duncan’s new multiple-range test (*p* < 0.05).

As with the exogenous ethylene, green peach aphid infestation also facilitated the EIN2C-YFP fusion protein localized in the nuclei of WT and *impβ1*/*IMPβ1-RFP* plants (Figure 5A). In WT and *impβ1*/*IMPβ1-RFP* plants that were not colonized with aphids, EIN2C-YFP was mostly located in ER membranes, instead of nuclei (Figure 5A). The nuclear localization of EIN2C-YFP was not detected in *impβ1* mutant plants with and without aphid colonization (Figure 5A). Taken together, these analyses elucidate that IMPβ1 transports EIN2C into the nucleus in response to aphid colonization in Arabidopsis.

The aphid-induced nuclear import of EIN2C in the presence of a functional IMPβ1 provides the molecular basis for IMPβ1 to regulate EIN2-dependent insect-deterrent defense responses, which mainly include callose deposition and PBD response gene expression [10,21,22]. We found that these responses were induced in coincidence with constitutive expression of the *EIN2C* gene and aphid-induced expression of the *IMPβ1* gene and the innate *EIN2* gene in *P35S:EIN2C:YFP*-transformed WT and *impβ1*/*IMPβ1-RFP* plants. In both plants, the expression of *IMPβ1* was significantly increased by artificial colonization with aphids in contrast to the ready-state expression levels detected in the aphid-free control (Figure 5B). Aphid-induced expression of the *EIN2C* was significantly inhibited in *impβ1* plants compared with WT and *impβ1*/*IMPβ1-RFP* plants colonized with aphids (Figure 5B).

In leaves of the WT and *impβ1*/*IMPβ1-RFP* plants, but not in the *impβ1* mutant, infestation with green peach aphid induced strong expression of EIN2-dependent insect-deterrent defense response genes (Figure 5C). The *PDF1.2* gene is a molecular marker of the ethylene-mediated insect-deterrent plant-defense mechanism [41]. The expression of *PDF1.2* is subject to ethylene signaling and provides a broad spectrum of resistance to herbivores, including leaf-eating and sap-sucking insects [11,42]. In contrast, the PBD response genes *PP2-A1*, *PP2-A2*, and *GSL5* are more specific in function against phloem-feeding insects, typically including aphids [21,22,25,28,41,43]. The aphid-induced expression of these PBD response genes (Figure 5C) coincided with enhanced callose deposition in leaves of the plants carrying a functional *IMPβ1* (Figure 5D,E). Taken together, these results suggest that the PBD activation is dependent on IMPβ1.

### 2.6. IMPβ1-Conferred PBD Inhibits Phloem Feeding and Massive Infestation by Aphids

**Figure 6:**
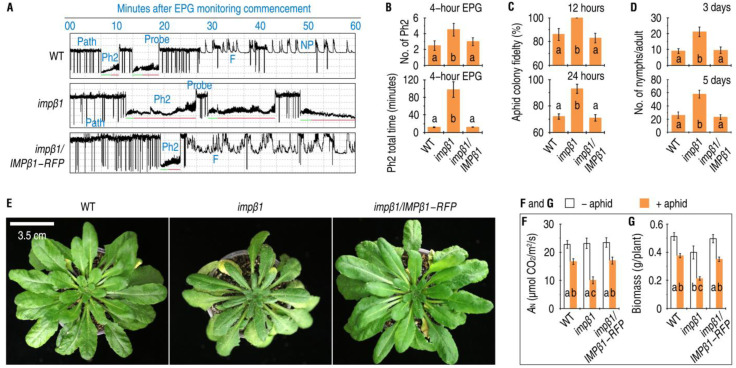
IMPβ1 contributes to Arabidopsis resistance against phloem-feeding activities and massive infestations by aphids. (**A**) The 2 h records from 4 h EPG monitoring of aphid feeding on leaves of the different plants. Uniform aphids were placed on upper surfaces of leaves living on 30-day-old plants and monitored with the EPG device. The EPG waveform patterns shown here represent feeding behaviors of eight aphids placed on each of the three plant genotypes. The non-puncturing phase (NP) indicates that the stylet remained outside the cuticle. Cell puncturing (probe) usually leads to the pathway phase (path) in which the stylet penetrates between cells en route to the vascular tissue [45]. A successful path navigates the stylet to the phloem phase (Ph2) to absorb the phloem sap. In order to prevent protein clogging inside the sieve element, E1 salivation (green lines) first ejects watery saliva [45,46]. Second, E2 saliva (red lines) is added to the ingested sap, thought to prevent phloem proteins, mainly phloem protein 1 (PP1) and phloem protein 2 (PP2), from clogging inside the capillary food canal [44]. During the feeding process, mechanical problems with stylet penetration into the plant tissues, namely, derailed stylet mechanics shown as F, may occur due to a deficient saliva composition [22,47]. Both path and F delay the time to the Ph2 and prevent ingestion of phloem sap [47]. Following a smooth Ph2, the xylem phase (XP) may proceed while aphids try to suck water from the xylem [10,25] to reduce osmotic pressure caused by increased sucrose concentrations in ingested phloem sap [44]. (**B**) Summation of Ph2 time as mean values ± SDs of results from three independent experiments, each involving eight aphids (*n* = 24 aphids). (**C**) Aphid colony fidelity (means ± SDs, *n* = nine biological repeats). (**D**) Aphid productivity rates (means ± SDs, *n* = nine biological repeats). (**E**) Symptoms of aphid infestation in the different plants 10 days after artificial colonization. Each photo represents nine plants. (**F**) Leaf net photosynthesis rate (*A*_N_) measurements (means ± SDs, *n* = six leaves). (**G**) Plant biomass (means ± SDs, *n* = 15 plants). In F and G, 30-day-old plants were colonized with or remained free from aphids and the measurements were performed two weeks later. In (**B**–**D**,**F**,**G**), the different letters on the graphs indicate significant differences, as assessed using Duncan’s new multiple-range test (*p* < 0.05).

The PBD induced by green peach aphid began to impede aphid feeding activities on Arabidopsis (Figure 6A). Feeding from plants by sap-sucking insects undergoes several phases that can be monitored by an electrical penetration graph (EPG) instrument in real time as distinct waveforms [21,22,25,44,45]. EPG-monitoring over 4 h (Supplementary Table S4) showed that green peach aphid feeding did undergo these major phases (Figure 6A), and that the aphids fed longer and more often from the phloem of the *impβ1* mutant than from the WT and *impβ1*/*IMPβ1-RFP* plants (Figure 6B). Over 4 h, the aphids fed in the phloem phase (Ph2) for a total of 97.4 ± 17.7 min from the *impβ1* mutant, but only 11.8 ± 0.8 min on WT and 4.3 ± 0.3 min on the complemented line.

Assessment of aphid colony fidelity (Figure 6C) and reproductivity rates (Figure 6D) showed that the WT and *impβ1*/*IMPβ1-RFP* plants were more resistant than the *impβ1* mutant to infestation by the aphid. Colony fidelity is the number of aphids remaining in the leaf colonies within 24 h, while reproductivity rate is the number of nymphs produced by an adult within five days after artificial colonization [21,22]. The aphid colony fidelity (Figure 6C) and reproductivity rates (Figure 6D) were significantly increased in the *impβ1* mutant compared with the WT and *impβ1*/*IMPβ1-RFP* plants. Over 24 h, the aphid fidelity averaged 72% in the WT, 71% in *impβ1*/*IMPβ1-RFP,* and 92% in *impβ1* plants, with about 22% higher in the mutant. In five days, totally 26, 23, and 58 nymphs, on average, were produced by an aphid adult individual in leaf colonies of the WT, *impβ1*/*IMPβ1-RFP,* and *impβ1* plants, respectively. In other words, the presence of a functional *IMPβ1* gene in the plants provided >55% resistance against aphid population growth. As a result of altered feeding, colonizing, and reproductive behaviors of the aphids, the *impβ1* mutant incurred more severe infestations and displayed leaf chlorosis and necrosis (Figure 6E). The leaf infestations by aphids caused substantial reductions in net photosynthesis rates shown as *A*_N_ variant (Figure 6F) and plant biomass (Figure 6G). These results demonstrate the critical role of IMPβ1 in the establishment of aphid-induced PBD, which develops in relevance to aphid-induced nuclear import of EIN2, impedes phloem-feeding activities, and reduces infestation of the plant.

### 2.7. EIN2C Complements the ein2-1 Mutant in PBD Responses

**Figure 7:**
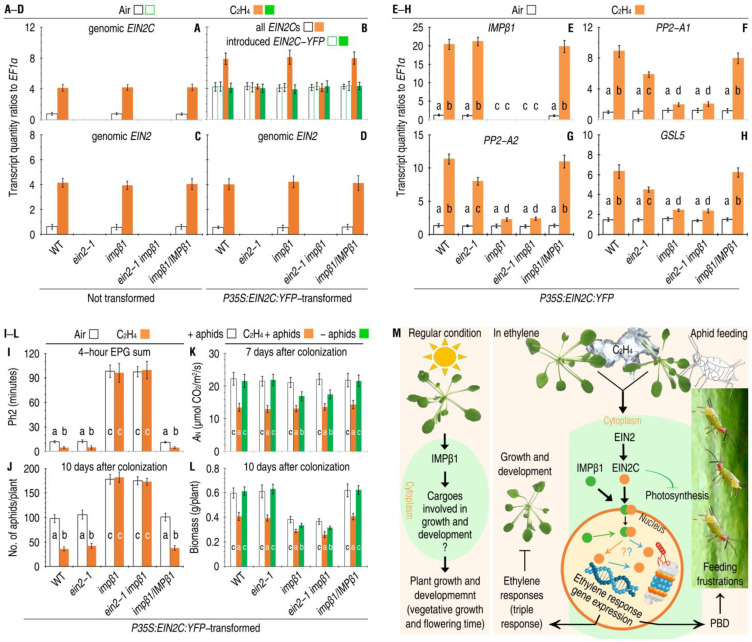
IMPβ1-mediated nuclear import of EIN2C is indispensable to EIN2-regulated PBD activation and is a crucial component in the proposed model of IMPβ1 functions. (**A**–**H**) EIN2C produced by transform expression complements the *ein2-1* mutant in PBD-defense-response gene expression. Leaves of the indicated plant genotypes were transformed or not. These plants were incubated in air or in 10 µL/L ethylene. Two days later, expression of the indicated genes in the transformed leaves was analyzed by qRT-PCR. (**I**–**L**) The combined effects of ethylene and EIN2C on phloem feeding and massive infestations of aphids and plant photosynthesis and growth. Plants were treated similarly as in (**B**). The displayed paragraphs were scored at the indicated times. (**A**–**L**) Data shown are mean values ± SDs, and the different letters on the graphs indicate significant differences, as assessed using Duncan’s new multiple-range test (*n* = 6, *p* < 0.05). (**M**) Model of IMPβ1 functions in plant growth and defense regulation. IMPβ1 at least has three functions depending on plant growth conditions. First, in plants growing under regular conditions, IMPβ1 participates in vegetative growth, possibly by targeting specific cargoes related to growth and development, but this hypothesis remains to be verified. Second, in plants treated with ethylene or incurring aphid infestations, IMPβ1 mediates the nuclear entry of EIN2C to confer ethylene response. Third, IMPβ1-guided nuclear import of EIN2C is also critical for activation of PBD, which effectively inhibits the phloem-feeding activities and massive infestations of aphids on the plants. Question marks (in the nucleus) indicate unanswered questions, namely, to what extent EIN2C is associated with the regulation of expression of ethylene-regulated transcription factors and defense genes [2,3,5,7,11,28,31,48], or with the proteasome activities that degrade ethylene-signaling repressors to facilitate signal transduction [14,21,22,25,28,33,34,49].

To look for the functional connection between IMPβ1-mediated nuclear import of EIN2C and ethylene-induced EIN2-regulated PBD responses, *EIN2C* was transformed in plants of the Arabidopsis WT, *impβ1*/*IMPβ1-RFP*, *ein2-1,* and *impβ1* single mutants, and *ein2-1 impβ1* double mutant. These plants were transformed with *P35S*:*EIN2C*:*YFP* or remained untransformed in control and then incubated in air and in 10 µL/L ethylene, respectively. Analyses by qRT-PCR performed 24 h later of the ready-state and ethylene-induced expression of the genomic *EIN2C* (Figure 7A) and total expression of the genomic and introduced *EIN2C* fragments (Figure 7B) in untransformed and transformed WT, *impβ1*, and *impβ1*/*IMPβ1-RFP* plants. The qRT-PCR data also confirmed the ready-state and ethylene-induced expression of the genomic *EIN2* in untransformed (Figure 7C) and transformed (Figure 7D) plants of WT, *impβ1*, and *impβ1*/*IMPβ1-RFP*. In particular, the introduced *EIN2C* was well expressed in the transformed plants of the *ein2-1* and *ein2-1 impβ1* mutants as in the other genotypes (Figure 7B).

Foliar expression levels of *IMPβ1* and PBD response genes were quantified. In the absence of ethylene, the expression of *IMPβ1* (Figure 7E) and PBD response genes, including *PP2-A1*, *PP2-A2*, and *GSL5* (Figure 7F–H), remained around the steady-state levels all plants. In the presence of ethylene, *P35S*:*EIN2C*:*YFP*-transformed WT, *impβ1*/*IMPβ1-RFP*, and *ein2-1* plants well supported the PBD-response gene expression (Figure 7F–H). However, ethylene displayed higher extents to induce the gene expression in the WT and *impβ1*/*IMPβ1-RFP* plants than in the *ein2* mutant (Figure 7F–H). Furthermore, the gene expression was considerably inhibited in the *impβ1* single mutant and *impβ1 ein2-1* double mutant, even in the presence of ethylene (Figure 7F–H). These differences suggest that transient constitutive expression of *EIN2C*:*YFP* temporarily compensates the *ein2-1* mutant defects in sufficient expression of the PBD-response genes only when ethylene and a functional *IMPβ1* are concomitantly present in the plants. This compensation caused strong inhibitions to the phloem-feeding activities of aphid (Figure 7I), resulting in marked reductions in the aphid infestations (Figure 7J) in *ein2-1* plants incubated with ethylene in contrast to the incubation in air. In this mutant, the introduced EIN2C provided inhibitions to aphid feeding and population growth as strong as in the WT and *impβ1*/*IMPβ1-RFP* plants (Figure 7I,J). On the contrary, the engineering *EIN2C* introduced into the *impβ1* and *impβ1 ein2-1* plants failed to execute any inhibitory effects on aphids, indicating that EIN2C functions downstream of IMPβ1 in ethylene signal transduction towards defense responses.

While aphid infestations substantially reduced leaf photosynthesis rates, photosynthesis was further inhibited by the exogenous ethylene and introduced EIN2C, as evidenced by variations in the *A*_N_ values detected from the plants with and without ethylene treatment, EIN2C introduction, and aphid infestations (Figure 7K). In contrast, *IMPβ1* did not evidently affected photosynthesis, as evidenced by equivalent *A*_N_ levels in the plants that carry or lack a functional *IMPβ1* (Figure 7K). While growth of the *impβ1* mutant was considerably impaired as compared with that of the WT plant, growth inhibition was further caused by the exogenous ethylene (Figure 7L). These analyses suggest that de novo expression of *EIN2C* can complement the *ein2-1* mutant also in photosynthesis and vegetative growth in addition to PBD responses.

## 3. Discussion

This study has focused on the function of IMPβ1 in nuclear import of EIN2C linked to plant PBD against aphid infestations (Figure 7M), a distinct model of biological interactions characterized by stylet penetration of plant cells and the cellularly specified responses to this unique stress on plant fitness [21,22,23,24,25,26]. Such a functional relationship between IMPβ1 and EIN2C is also recognized as a fascinating question for the central role that EIN2 bears in ethylene signal transduction towards plant growth, development, and immunity [1,3,4,5,6,7]. Now the scientific community commonly appreciates that EIN2C liberation from the full-length EIN2 sequence and subsequent trafficking to the nucleus represent pivotal events in the intricate ethylene signaling networks [2,7,9,12,13].

Since EIN2^454–1294^ was demonstrated to be a sufficient regulator of ethylene signaling in Arabidopsis [1], many EIN2C fragments have been characterized [7,12], and some fragments verified to regulate ethylene responses associated with plant growth and development [7,9,12,13,14,15]. Increasing studies have been devised to elucidate the functional relationship between subcellular localizations and biological performance of the length-varied EIN2Cs and even the full-length sequence of EIN2 in plants [7,9,12,13,14,15]. The present study extends the functional scope of the first identified EIN2C, namely, EIN2^454–1294^ [1], to plant defenses against insect infestations. Genetic, molecular, and cytological data obtained in this study clearly demonstrate that this EIN2C is navigated by IMPβ1 into plant nuclei to confer EIN2-dependent PBD responses under induction either by the exogenous ethylene or by aphid infestation (Figure 7M). Especially, EIN2C can complement the *impβ1* mutant, restoring it to the WT in EIN2-dependent PBD responses. Considering these findings, researchers in the scientific community would not find it difficult to comprehend that via nuclear import of EIN2C, IMPβ1 are imported via nuclear transport for regular plant growth and development, physiological responses, and immunological responses, respectively (Figure 7M). IMPβ1 may be a constituent of normal growth and development (Supplemental Figure S1B), at least affecting vegetative growth. The transition to flowering marks a key adaptive developmental switch in plants which has an impact on their survival and fitness. It is necessary to further identify any other physiological processes affected by IMPβ1. It is particularly necessary to characterize the functional relationship between IMPβ1 or IMPβ1-guided EIN2C trafficking and any regulators of the floral transition pathways that interplay with plant hormones to determine flowering time [50].

Explaining the relationship between subcellular localization and biological performance of EIN2Cs and full-length EIN2 in plants has been a fascinating problem. The different EIN2C species were found in association with the ER, in the nucleus, or over the cytoplasm [9]. By studying five EIN2Cs, either localizing them to the ER membrane or associating them with the nuclear fraction, Wen and colleagues proposed that the ethylene signal promotes the cleavage of the C-terminal portion from ER-located EIN2, and facilitates its nuclear localization to stabilize the EIN3 protein [9]. They determined that the nuclear localization of the EIN2Cs is sufficient to activate EIN3-mediated transcription and ethylene responses [9,51]. Similar results were obtained by Zhang and colleagues. They determined that an EIN2C was cleaved from EIN2 and moved into the nucleus to facilitate jasmonate-induced leaf senescence [15]. Zhang and colleagues proposed “an alternative model of ethylene signaling” [7]. In this model, EIN2 prevented from activation is targeted by its interacting proteins to the 26S proteasome to be degraded in the absence of ethylene; in the presence of ethylene, EIN2 is released from the inhibition to activate ethylene signaling by trafficking from the ET to the nucleus, while EIN2C species assist EIN2 trafficking and the subsequent ethylene responses [7]. Our study demonstrating that IMPβ1 mediates EIN2C entry to the nucleus in response to ethylene treatment or aphid infestation agrees with the recently proposed “alternative model of ethylene signaling”.

IMPβ1-mediated EIN2-dependent insect-deterrent responses characteristic of PBD effectively impede massive infestations of aphids in Arabidopsis, indicating the potential value of EIN2C in protecting crops from insect attacks. Coincidently, evidence [52,53,54,55] has shown that some physiological regulators function not only in growth and development but also in defense responses, possibly bridging the problem of growth–defense tradeoffs, or the costs to fitness that accompany defense responses [26,55,56]. While nucleocytoplasmic trafficking is the most important transportation route inside plant cells, the mechanisms supporting this trafficking need further exploration [52,53,54,55,56]. The functions of IMPβ1 in EIN2C trafficking link plant growth and defense regulation (Figure 7M) now offer a mechanism that could be used to bridge the growth-defense dichotomy. These molecules could be used to concomitantly improve both defense and productivity in plants, especially crops [55,57]. Since the *impβ1* mutant is compromised in both growth and defense, *IMPβ1* loss-of-function has negative consequences for both sets of processes, rather than creating a trade-off between them. Future studies will focus on engineering the overexpression of *IMPβ1* homologs in crops with the aim of synchronized enhancements of crop productivity and immunity.

## 4. Materials and Methods

### 4.1. Plant Material and Growth Conditions

Arabidopsis ecotype Col-3, the *ein2-1* single mutant, and 25 *imp* mutants were previously generated in the Col-3 background. Their seeds were purchased from TAIR (The Arabidopsis Information Resource at www.arabidopsis.org accessed on 12 March 2009). The *ein2-1 impβ1* hybrid and *impβ1*/*IMPβ1-RFP* transgenic Col-3 lines were generated in the HD lab and used in F6-selfing homozygous generation in this study. Seeds were germinated in flat plastic trays filled with a plant growth substrate. Three days later, germinal seedlings were moved into Φ7-cm pots (1–3 plants per pot) filled with the same substrate. Seed germination and plant growth were accommodated in environmentally controlled plant growth chambers under 24 ± 1 °C, 250 ± 50 μmol quanta/m^2^/s illumination, and a photoperiod circle of 8 h light and 16 h dark.

### 4.2. Aphid Cultures

A single isolate of *M. persicae* was collected from field-grown radish (*Raphanus sativus*), near Nanjing in China. A clone of apterous agamic females was obtained by acclimatization in WT Arabidopsis grown in the chamber. The subsequently formed colonies were maintained in nursery Arabidopsis seedlings and were transferred to fresh plants every two weeks in the HD lab located at the Nanjing Agricultural University Weigang Campus (2000–2019) and the Shandong Agricultural University North Campus (since 2018). Uniform 10-day-old aphids were used in this study and were transferred to experimental plants with a fine paintbrush.

### 4.3. Plant Colonization

For Arabidopsis colonization, uniform 10-day-old *M. persicae* aphids were placed on the top two expanded leaves of plants (10 aphids per leaf). A total of 1200 aphids were monitored in six independent experiments for each genotype and for each single combination of treatment and plant genotype. For each treatment, 200 aphids were placed on 20 leaves (two leaves on 10 plants). Aphid movement from leaf colonies was monitored for five days, and the number of aphids in a leaf colony was scored at 24 h intervals [21,22,25]. The number of nymphs that moved away from colonies was also counted. The proportion of aphids staying in a leaf colony was regarded as colony fidelity. Aphid reproduction was surveyed twice a day by counting newborn nymphs. The reproduction rate was quantified as the ratio between the total number of nymphs produced in five days and the total number of aphid adults that stayed in leaf colonies during the same period [21,22,25].

### 4.4. Gene Expression and PBD Analyses

Total RNA was extracted via the RNA-easy Isolation Reagent Kit (Vazyme, R701-01, Nanjing, China). Plant gene expression was quantified by real-time quantitative reverse transcriptase polymerase chain reaction (qRT-PCR) performed under strict experimental designs (Supplementary Table S2). All the qRT-PCR analyses were conducted using primers (Supplementary Table S3) of high specificities, which were verified by the melting-curve method [22], and using the constitutively expressed *EF1α* gene as a reference [10]. The qPCR experiments were conducted on the ABI QuantStudio 3a 96 Real-Time PCR system (Thermo Fisher, Waltham, Massachusetts, USA) using the ChamQ^TM^ Universal SYBR^®^ qPCR Master Mix (Vazyme, Q711-02, Nanjing, China). Relative expression levels of the tested genes (*IMPβ1*, *EIN2C*, related variants, and defense response genes, listed in Supplementary Table S3) were quantified as ratios of their transcript amounts to the *EF1α* transcript quantity. Callose visualization was performed on leaves as previously described [11].

### 4.5. Ethylene Treatment

Arabidopsis plants were treated with gaseous ethylene in 15 L glass vacuum chambers using the well-established protocol [58,59]. The container has a dome top cover with a valve in its center that allows vacuum removal of air and administration of ethylene. Before use, all interfaces of the individual components of the container were daubed with petroleum jelly to ensure complete sealing. For treatment, pots containing plants or agar plates containing seeds were placed into the glass container, the container was closed and some air was pumped out. Ethylene gas at the final concentration of 10 µL/L was injected into the container using a syringe and needle through the valve. After ethylene gas was pushed into the container, the valve was left open for a few seconds, allowing the outside air to enter the container so that the total air volume was brought back to the regular level. The valve was closed, and the container was moved into the plant growth chamber to incubate the plants or seeds. Untreated plants were placed in different vacuum containers, but ethylene was not applied.

### 4.6. Aphid Feeding Behavior Monitoring

Aphid feeding activities were observed by the EPG technique [43,44] using the Giga Amplifier system (EPG Systems, Dillenburg, 12, 6703CJ, Wageningen, The Netherlands). Uniform 10-day-old aphids were placed on the upper side of the upper two expanded leaves of an Arabidopsis or wheat plant. The aphids were monitored in three independent experiments. Each experiment involved a total of eight aphids tested with one aphid per leaf using eight plants. Immediately after aphids were placed on leaves, a 20-mm diameter gold wire was attached to the dorsal surface of each aphid’s abdomen using silver conductive paint. The other end of the wire was connected to an eight-channel Giga-8 direct current amplifier with eight channels and a 10^9^-Ω input resistance in an electrical circuit that is also connected to the plant via an electrode placed in the soil. The behavior of individual aphids was monitored for 4 h. Voltage waveforms were digitized at 100 Hz with an A/D converter USB device. Waveform patterns were identified according to previously described categories [25,44]. Briefly, the nonpuncturing phase (NP) indicates the stylet is outside the cuticle. Cell puncturing (probe) leads to the pathway phase (Ph2) in which the stylet penetrates between cells en route to the vascular tissue [21,22,25,45]. When the phloem is not a favorite source for feeding, the xylem phase (XP) may be observed while aphids try to suck sap from the xylem [21,22,25].

### 4.7. Gas Exchange Measurements

Gas exchange in the second and third leaves from the top of plants was measured with the LI-6800 photosynthesis system (LI-Corp Biosci, Lincoln, NE, USA). Detailed measurements on single leaves were performed following the manufacturer’s instructions and previously described experimental procedures [39,60]. During measurements, relative humidity in the leaf chamber (2 cm^2^ for Arabidopsis and 6 cm^2^ for wheat) was constantly maintained at 45% and the leaf temperature was kept at 25 °C. CO_2_ concentrations at the inlet and outlet of the leaf chamber were monitored by the non-dispersive infrared gas analyzer installed in the system. Photosynthetically active photon flux density was controlled by adjusting intensities of the lamp-house irradiation. Readings of *A*_N_ were documented automatically by the LI-6800 monitor system integrated into the LI-6800 system.

### 4.8. Genetic Complementation

The genetic complement was constructed in the plant binary vector pCAMBIA1301 [10]. The full-length sequence (1–4634) of the canonical WT *IMPβ1* gene was linked N-terminally with its own promoter sequence of nucleotides –2000 to –1 and linked C-terminally with the *RFP* gene sequence. Plant transformation with the recombinant vector and molecular characterization of transgenic lines were performed by conventional protocols [10,25] and T3 homozygous progenies were used in this study.

### 4.9. Protein-Protein Interaction Assays

As a first step, each of the four *IMP*s (*IMPα1*, *IMPα7*, *IMPβ1*, and *IPT*) were cloned into the pGADT7 bait vector, and EIN2C was cloned into the pGBKT7 prey vector of the Dualsystems’ split-ubiquitin yeast two-hybrid system (Dualsystems Biotech, Schlieren, Zurich, Switzerland). For YFP BiFC, previously constructed pCAMBIA1301-*YFP^N^* and *-YFP^C^* plasmid vectors were used in gene recombination. An *IMP* gene was fused to *YFP^N^* between the *Kpn*I and *Xba*I restriction sites, whereas *EIN2C* was linked to *YFP^C^* using the *Kpn*I and *Bam*HI recognition sites. Similar operations were used in luciferase assay except for replacing YFP^N^ and YFP^C^ with Luc^N^ and Luc^C^, respectively. The presence and absence of molecular interactions were determined using previously described protocols [34,39].

### 4.10. Subcellular Localization of IMPβ1 and EIN2C

The different chimeric genes were separately constructed in the plant binary vector pCAMBIA1031, transferred into GV3101-bacterial cells, and transiently expressed in plants using a previously described protocol [61]. Alternatively, plants were co-transformed with each of these constructs and the *IMPβ1*:*RFP* construct. 48 h after plant transformation, the EIN2C:YFP fusion protein in leaf cells was visualized by CLSM [10,57]. DAPI was used to stain nuclei and applied in an aqueous solution to immerse tested leaves 10 min before observation [61].

### 4.11. Statistical Analysis

Analysis of variance, student’s t-tests, and Duncan’s new multiple-range tests [62] were performed with GraphPad Prism 8.0 (https://www.graphpad.com/, accessed on 3 August 2019) to determine significance of differences in paired and multiple data from different plants or treatments.

## Data Availability

Not applicable.

## Supplementary Materials

The following supporting information can be downloaded at: https://www.mdpi.com/article/10.3390/ijms24108545/s1.
