# Supplementary material for: Importin β1 Mediates Nuclear Entry of EIN2C to Confer the Phloem-Based Defense against Aphids
Source: Int J Mol Sci. 2023 May 10;24(10):8545. doi: 10.3390/ijms24108545 (PMC10217940; doi:10.3390/ijms24108545)
Supplement: Supplementary file 1 [file ijms-24-08545-s001.zip › ijms-2339403-supplementary.pdf]

# Importin β1 mediates nuclear entry of EIN2C to confer the phloem-based defense against aphids

**Kai Lu[1], Liyuan Zhang[1], Lina Qin[1], Xiaochen Chen[1*], Xiaobing Wang[2], Meixiang Zhang[3], Hansong Dong[1*]**

## Supplementary material

**Supplementary Table S1** Information on Arabidopsis importins and insertional mutants found in The Arabidopsis Information Resource (TAIR) and National Center for Biotechnology Information (NCBI) databases

| NCBI code | Mutant seed stock no. | Gene symbol / gene product | Mutation (T-DNA insertion) region / site |
|---|---|---|---|
| At3G17340 | SALK_023981 | *PLmN* / protein import into nucleus | Promoter / 946 |
| | SALK_035684 | | Exon / 2433 |
| | SALK_119665 | | Exon / 5619 |
| At1G12930 | CS816806 | *IMP-R* / importin-related | Intron / 2214 |
| At1G26170 | SALK_096645 | *PTA-1* / protein transporter | Promoter / 473 |
| | CS819506 | | Promoter / 305 |
| | CS811453 | | Promoter / 324 |
| | SALK_062411 | | Intron / 5754 |
| At3G08947 | CS802995 | *PTA-2* / protein transporter | Promoter / 763 |
| At2G46520 | CS800336 | *IMPαER* / protein transporter | Promoter / 490 |
| At3G59020 | CS806403 | *IPT* / protein import into nucleus | Exon / 6318 |
| | SALK_069289 | | Exon / 6271 |
| | CS811646 | | Promoter / 193 |
| | CS806231 | | Promoter / 378 |
| | CS843329 | | Promoter / 202 |
| At5G53480 | CS817444 | *IMPβ1* / importin β1, putative; Other names: ATKPNB1, HOMOLOG OF HUMAN KPNB1, IMB1, KPNB1 | 3'-UTR / 3931 |
| At3G06720 | CS842229 | *IMPα1* / importin α1 | Promoter / 458 |
| At4G16143 | SALK_099707 | *IMPα2* / importin α2 | Exon / 1834 |
| At4G02150 | SALK_119474 | *IMPα3* / importin α3 | Exon / 2019 |
| At5G49310 | SALK_072939 | *IMPα5* / importin α5 | Intron / 267 |
| | SALK_023989 | | Exon / 1073 |
| | CS800857 | | Promoter / 170 |
| At3G05720 | SALK_020428 | *IMPα7* / importin α7 | Exon / 3660 |
| At3G03070 | CS812984 | *IMPα9* / importin α9 | Promoter / 251 |
| | SALK_005888 | | Promoter / 173 |

**Supplementary Table S2** Essential information on qRT-PCR experiments performed in this study

**Experimental design**

Plants in "treatment group" were artificially colonized with aphids or shifted into 10 µL/L ethylene, while plants in "control group" remained free from aphids and the exogenous ethylene application. Within each group, at least 90 plants were tested in 6 independent experiments (biological repeats), each involving 15 plants assigned into 3 repeats (technical repeats), except specified elsewhere.

**Sample**

In each experimental repeat, the topper second and third leaves were excised from at least 5 plants at the scheduled time point, weighted together, and quickly and softly placed into several 1.5-mL tubers with the amount of 200 mg per tube. The tubers were supplemented with proper volume of liquid nitrogen and homogenized immediately in a tissue homogenizer (Tissulyer-96, Shanghai Jingxing Co. LTD). The homogenates were immediately subjected to RNA extraction.

**Nucleic acid extraction**

RNA was isolated from the leaf homogenates by using RNA-easy Isolation Reagent (R701-01, Vazyme Biological and Medical Co LTD, Nanjing, China), resolved in DEPC-treated water, and quantified (to determine A260/A280 ratios) with a supper-microanalyzer, namely NanoDrop One C (Thermo Fisher). RNA preparations were used immediately or divided into 10-µL portions pouring into tubers, which were stored at -80°C and used in the subsequent experiments. Additional reagents and highly purified water used in RNA preparation and the subsequent operations were provided commercially as RNA-free products. All tools (such as tubers) used in RNA preparation and the subsequent operations were treated with DEPC, autoclaved at 125°C and 1.05 kg/cm$^2$ for 20 minutes, and cooled to room temperature before use.

**Reverse transcription**

Possibly existed genomic DNA residues were removed by HiScript® II 1st Strand cDNA Synthesis Kit (+gDNA wiper) from market (R212-02, Vazyme Biological and Medical Co LTD, Nanjing, China) a volume of 16-µL at 42°C for 2 minutes, containing 1 µg of RNA samples, 4-µL 4× gDNA wiper mix, 1-µL Oligo(dT)$_{23}$VN (50 µM), 1-µL Random hexamers (50 ng/µL) and RNase Free d$H_2O$. Immediately after this, first-strand cDNA synthesis was performed by adding 2-µL of 10× RT Mix and 2-µL HiScript II Enzyme Mix at 50°C for 15 minutes, 85°C for 2 minutes.

**qPCR target information**

Gene symbols, sequence accession numbers, and amplified length are provided in Supplementary Table S3. Every qPCR product was confirmed by sequencing and BLAST-aided comparison with the published sequence. All genes analyzed by qPCR were designed to amplify coding sequences, neglecting any possibilities of alternative splicing under any conditions.

**qPCR oligonucleotide**

Primer sequences and nucleotide modifications required for gene cloning, recombination, and/or base protection are provided in Supplementary Table S3.

**qPCR protocols**

***Reaction conditions***: The qPCR experiments were conducted on ABI QuantStudio 3 a 96 Real-Time PCR system (Thermo Fisher, USA) using ChamQ™ Universal SYBR® qPCR Master Mix (Vazyme, Q711-02, Nanjing, China), using a 20-µL reaction solution. This solution was made of 10-µL of 2× ChamQ Universal SYBR qPCR Master Mix, 0.4-µL 10 µM of each primer, 2-µL 10-fold dilutions of cDNA template, and DEPC-water. All the qPCR protocols were performed in triplicate on 96-well plates, along with blank (template-absent) control.

***PCR program:*** 95 °C, 30 seconds; 95°C, 10 seconds, 60°C, 30 seconds, 40 cycles.

**qPCR validation**

In initial experiments, four pairs of primers were designed for a single gene that was to be analyzed (or for every of tested genes). Each pair of the primers was assessed in the PCR program to amplify the gene cDNA at a concentration gradient, each loaded in a well of a plate. Using the same plate and PCR program, the reference gene (*EF1α* here) cDNA was simultaneously amplified, also at a concentration range, using the specific primers that had been verified to have a high amplification efficiency ($R^2$ = 0.99). For each pair of the primers designed for the tested

| |
|---|
| gene, the Log10-dilution curve was established with the GraphPad Prism 8 software mounted into the thermometer, the $R^2$ value was automatically shown on the monitor. If any of the 4 primers pairs had $R^2$ value higher than 0.98, it was subjected to confirmative assessment by the automatic melting-curve program. A melting curve was obtained by slow heating from 60°C through 95°C at a 0.1°C elevation per second, and by continuous fluorescence collection during the amplification process. A desired primer pair had a sharp derivative peak between 80 and 90°C. If a prime pair was verified by the Log10-dilution curve and melting-curve methods in initial experiments, and if it was used under the same experimental conditions, only the melting curve was evaluated. |
| **Data analysis**<br>The protocol-detected mRNA expression of a tested gene was normalized against that of *EF1α*. Briefly, the qPCR program was analyzed by Quant Studio™ Design & Analysis Software v1.4.3 coupled with the thermometer. The relative quantity of a tested gene transcript was calculated using the $2^{-\Delta\Delta Ct}$ method and given as the ratio the *EF1α* transcript amount. The qRT-PCR data were analyzed statistically as described in the main text and Figure legends. |

## **Supplementary Table S3** Information on genes tested and primers used in this study

| Gene | Locus code (source) | Primers / product length (bp) / subjects |
|---|---|---|
| *AtIMPβ1* | At5G53480 | 5’-GCTCTAGAATGGCAATGGAGGTTACGCAG-3’, 5’-CCGGAATTCAACAGATATGGCACGGGTTATG-3’ / 2610 / cDNA cloning and recombination |
| | | 5’-GCCTAATGTTGCCGAGAAGG -3’, 5’-GCGTGATTCAGTTGCATCCT-3’ / 154 / qRT-PCR |
| *AtEIN2* | AT5G03280 | 5’-CTTATGCCTGATACTCTGGCT-3’, 5’-CCGCACTATTGATTCGTCT-3’ / 172 / qRT-PCR |
| *AtEIN2C* (EIN2$^{454–1294}$) | AT5G03280 | 5’-GCTCTAGACTCTGGCTGGCAGCCACGCC-3’, 5’-CCGGAATTCACCCAATGATCCGTACGCAGTC-3’ / 2523 / cDNA cloning and recombination |
| | | 5’-AGCCAGGGCAAAGAAACT-3’, 5’-TGTCCTCTGCTGCTTCAGT-3’ / 178 / qRT-PCR |
| *RFP* | KY379159.1 | 5’-AACAGATATGGCACGATGGCCTCCTCCGAGAACGT-3’, 5’-AACTGCAGCAGGAACAGGTGGTGGCGGCCC-3’ / 675 / cDNA cloning and recombination |
| *YFP* | AY818378.1 | 5’-ACCCAATGATCCGTAATGGTGAGCAAGGGCGAGGA-3’, 5’-AACTGCAGGTACAGCTCCTCCATGCCGA-3’ / 714 / cDNA cloning and recombination |
| *AtEF1α* | AT5G60390 | 5’-CACGAGTCTCTTCTTGAG-3’, 5’-GGATCATCCTTGGAGTTAG-3’ / 113 / qRT-PCR |
| *AtIMPβ1-RFP* | At5G53480+ KY379159.1 | 5’-GTGGGTGGTTTGATACAACAG-3’, 5’-ATGAACTCGGTGATGACG-3’ / 164 / qRT-PCR |
| *AtEIN2C-YFP* | AT5G03280+ AY818378.1 | 5’-CCGCAGTCACACTTCTTGA-3’, 5’-CTTGCTCACCATACCCAA-3’ / 236 / qRT-PCR |
| *AtPDF1,2* | AT5G44420 | 5’-AGAAGTTGTGCGAGAAG-3’, 5’-TGTGCTGGGAAGACATAG-3’ / 127 / qRT-PCR |
| *AtPP2-A1* | AT4G19840 | 5’-AGTCTTGCCTAACGGTAAGG-3’, 5’-TTCTTCCAAACACCAGCC-3’ / 168 / qRT-PCR |
| *AtPP2-A2* | AT4G19850 | 5’-GGCGAGAGAATGTGAACCT-3’, 5’-CAATAAGACCAGACTTCCACTG-3’ / 147 / qRT-PCR |
| *AtGSL5* | AT4G03550 | 5’-GTCGCATTGTTATGGTTGC-3’, 5’-AGTTGTTCCTCAGGCATTAGG-3’ / 200 / qRT-PCR |
| *TaHfr-1* | AF483596 | 5’-AGCATCCCTCACCATTA-3’, 5’-GCACGAAAGAAACCCAC-3’ / 118 / qRT-PCR |
| *TaWci-1* | U32427 | 5’-CCAGCGAACGGAGGTT -3’, 5’-CCAGTCATTAGCAGCAAGG-3’ / 130 / qRT-PCR |
| *TaGSL2* | DQ086483 | 5’-CTGGGAGTGCTGGTGCTGAT-3’, 5’-TAACAATCACTCCAAGCAGTATCTC-3’ / 208 / qRT-PCR |

| | | |
|---|---|---|
| *TaGSL10* | DQ086486 | 5'-GGGGACAGAGCGAGAACA-3', 5'-GTAGGCAACACGAAGAGC-3' / 193 / qRT-PCR |
| *TaGSL12* | DQ086487 | 5'-GTTCTTCTCGTGGTTCCCCTTT-3', 5'-GTCCCTAATCAAGTCCAGAAATGTA-3' / 182 / qRT-PCR |
| *AtPlmN* | At3G17340 | 5'-TCCTGTGCCTTCGAAGAGTT-3', 5'-AAATGGGTAGCACACGGGTA-3' / 152 / qRT-PCR |
| *AtIMPR-R* | At1G12930 | 5'-TGCCCTTGAGGGAGATTGAG-3', 5'-TCAAAGGCCTCTCCCTCTTG-3' / 82 / qRT-PCR |
| *AtPTA-1* | At1G26170 | 5'- TACGCTGGTACCTGTGTTGT-3', 5'- CACAGGCGTCACCAAAGTAG-3' / 172 / qRT-PCR |
| *AtPTA-2* | At3G08947 | 5'-TGCTGCGTATGAAACCTTG-3', 5'-AACACCACACAGAGAAGCCT-3' / 181 / qRT-PCR |
| *AtIMPαER* | At2G46520 | 5'-ATCCAGAGCCAGTTGAGTG-3', 5'-CGGTCCCAAGAATACCATT-3' / 151 / qRT-PCR |
| *AtIPT* | At3G59020 | 5'-AACTTGGCCAGTGCTTTCTG-3', 5'-CCAATGCACCAGCATCATCA-3' / 76 / qRT-PCR |
| *AtIMPα1* | At3G06720 | 5'- GGGTCACACAGGAGACATGA-3', 5'- GCCTGAGAACCATCAACACC-3' / 192 / qRT-PCR |
| *AtIMPα2* | At4g16143 | 5'-CCACTTCAGGTGGTTCTCCT-3', 5'-AATCCTTGGATCAGGGCACA-3' / 98 / qRT-PCR |
| *AtIMPα3* | At4G02150 | 5'-CTCGCCTCATCCAGCTCTTA-3', 5'-TTGCTGGTCGAGAACCATCT-3' / 119 / qRT-PCR |
| *AtIMPα5* | At5G49310 | 5'-TTACCCGTTCTTGCTGACCT-3', 5'-TTGTGCCAGATTGACCAACG-3' / 156 / qRT-PCR |
| *AtIMPα7* | At3G05720 | 5'-AACGCCGCTTGGACTTTATC-3', 5'-CACAAACATTGGCTTCAATCAC-3' / 202 / qRT-PCR |
| *AtIMPα9* | At5g03070 | 5'-GCGGGACAAAGAAGACGAAA-3', 5'-AGTACCCACCCTACAAAGCC-3' / 93 / qRT-PCR |

**Supplementary Table S4** Four-hour EPG analyses of the green peach aphid feeding from Arabidopsis plants

| Feeding behaviors | | Wild type | *impβ1* | *impβ1/IMPβ1-RFP* |
|---|---|---|---|---|
| Number of nonpuncturing phase | total | 27.2 ± 5 | 4 ± 0.8 | 6.2 ± 1.3 |
| | 1st h | 9 ± 2 | 4 ± 0.8 | 2 ± 0.1 |
| | 2nd h | 8 ± 2 | 0 | 0 |
| | 3rd h | 7 ± 0.8 | 0 | 2 ± 0.5 |
| | 4th h | 3 ± 0.1 | 0 | 2 ± 0.5 |
| Duration of nonpuncturing, minutes | total | 47.7 ± 8.8 | 9.2 ± 1.3 | 2.8 ± 0.7 |
| | 1st h | 15.2 ± 2 | 9.2 ± 1.3 | 1.1 ± 0.3 |
| | 2nd h | 7.2 ± 1.2 | 0 | 0 |
| | 3rd h | 20 ± 5 | 0 | 0.7 ± 0.1 |
| | 4th h | 5.2 ± 0.5 | 0 | 0.8 ± 0.1 |
| Time to 1st cell puncturing, minutes | | 15 ± 1.3 | 5 ± 0.9 | 14.8 ± 1.3 |
| Time to 1st pathway, minutes | | 19.2 ± 1.8 | 10.2 ± 1.2 | 14.2 ± 1.1 |
| Number of pathway phase | total | 17.2 ± 2.4 | 10.2 ± 2 | 22.4 ± 3.9 |
| | 1st h | 4 ± 0.5 | 4 ± 1 | 4.2 ± 0.5 |
| | 2nd h | 6 ± 1.2 | 3 ± 0.3 | 5 ± 0.7 |
| | 3rd h | 0 | 2 ± 0.5 | 5 ± 0.6 |
| | 4th h | 7 ± 0.5 | 1 ± 0.1 | 8 ± 2 |
| Duration of pathway phase, minutes | total | 47 ± 3 | 67.7 ± 8 | 92.3 ± 9.9 |
| | 1st h | 13.8 ± 0.5 | 19.2 ± 2 | 20.6 ± 2.5 |
| | 2nd h | 16.7 ± 2 | 18.3 ± 1.9 | 20.8 ± 2.3 |
| | 3rd h | 0 | 23.3 ± 2.8 | 35 ± 3 |
| | 4th h | 16.3 ± 0.3 | 6.7 ± 1.1 | 15.9 ± 2.1 |
| Time to 1st phloem phase, min | | 66.6 ± 10 | 71.6 ± 6.7 | 79.2 ± 8 |
| Number of cell puncturing after 1st phloem phase | | 17 ± 3 | 3 ± 0.3 | 18 ± 3.5 |
| Number of phloem phase | total | 2.5 ± 0.6 | 4.5 ± 0.8 | 2.6 ± 0.2 |
| | 1st h | 0 | 0 | 0 |
| | 2nd h | 2 ± 0.3 | 2.5 ± 0.5 | 1 ± 0.1 |
| | 3rd h | 0 | 1 ± 0.1 | 0 |
| | 4th h | 0.5 ± 0.2 | 1 ± 0.1 | 1.5 ± 0.1 |
| Duration of phloem phase, minutes | total | 11.8 ± 0.8 | 97.4 ± 17.7 | 11.3 ± 0.3 |
| | 1st h | 0 | 0 | 0 |
| | 2nd h | 10.6 ± 0.5 | 39.2 ± 7.1 | 4.2 ± 0.2 |
| | 3rd h | 0 | 8 ± 1.5 | 0 |
| | 4th h | 1.1 ± 0.1 | 50 ± 9 | 7.1 ± 0.1 |
| Duration of salivation, minutes | total | 11.8 ± 0.8 | 97.4 ± 17.7 | 4.3 ± 0.3 |
| | E1 | 2.7 ± 0.4 | 22.4 ± 3.8 | 1.3 ± 0.1 |
| | E2 | 9.1 ± 0.3 | 75 ± 13.6 | 3 ± 0.2 |
| Number of derailed stylet mechanics | total | 1 ± 0.2 | 1 ± 0.2 | 6 ± 1.1 |
| | 1st h | 0 | 0 | 3 ± 0.5 |
| | 2nd h | 0 | 0 | 3 ± 0.5 |
| | 3rd h | 1 ± 0.2 | 1 ± 0.2 | 0 |
| | 4th h | 0 | 0 | 0 |
| Duration of derailed stylet mechanics, minutes | total | 3.3 ± 0.2 | 5.8 ± 0.7 | 25.5 ± 4.5 |

## Supplementary Figures

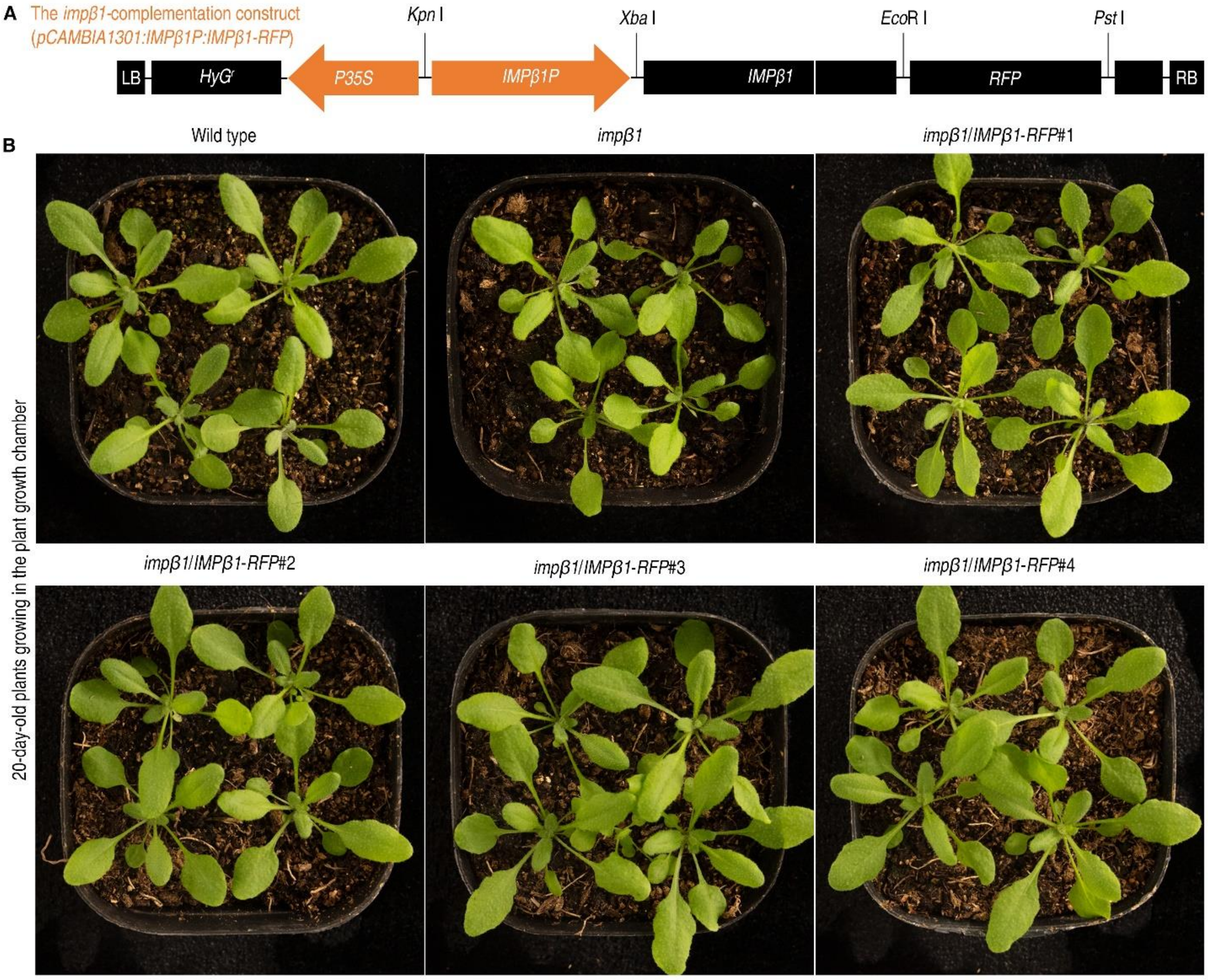


**Supplementary Figure S1.** *IMPβ1* promoter fused to *IMPβ1* transgenic *impβ1* mutant. **(A)** Diagram of the genetic complementation construct essentially made of the *IMPβ1* gene promoter (*impβ1P*) and coding sequence (*IMPβ1*) fused to the *RFP* gene encoding red-fluorescent protein (RFP). **(B)** Plants photographed at vegetative stages during growth under 15 days. Each image represents 18 leaves from 9 plants investigated in 3 independent experiments.

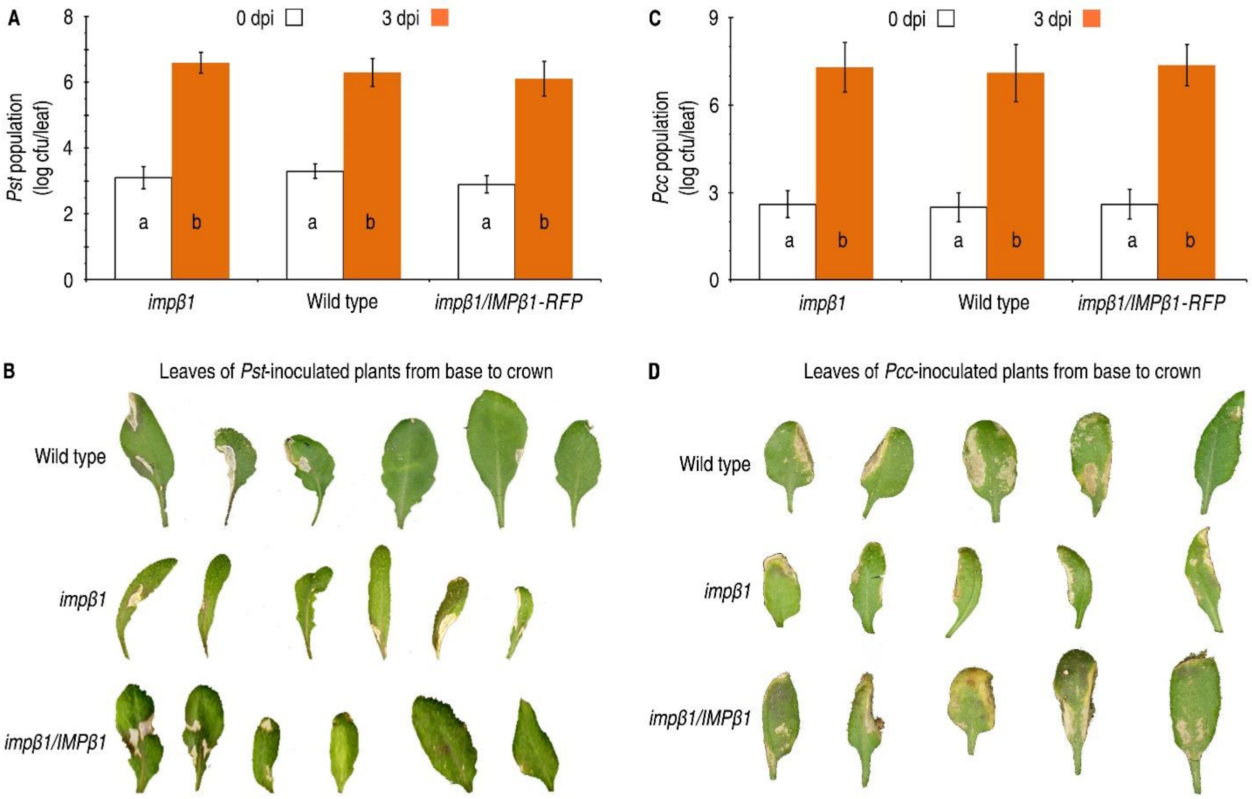


**Supplementary Figure S2.** Virulence of two bacterial pathogens on the different Arabidopsis genotypes with and without a functional *IMPβ1*. **(A)** *Pseudomonas syringae* pv. *tomato* (*Pst*) population quantified as colony formation unit (cfu) after recovery from leaves 0 dpi (day post-inoculation) and 3 dpi. **(B)** *Pst*-caused leaf symptoms, which were photographed at 10 dpi, leaves are representative of 90 plants. **(C)** *Pectobaterium carotovora* subsp. *carotovora* (*Pcc*) population determined with data shown as in A. **(D)** *Pcc*-caused leaf symptoms, which were photographed at 10 dpi and are representative of 90 plants. In **(A** and **C)**, data shown are mean values ± SDs. Different letters on the graph indicate significant differences of data in multiple comparison by ANOVA and Ducan's new multiple range test ($n$ = 6 independent experiments each involving 15 plants tested in 3 biological repeats, $P < 0.05$).

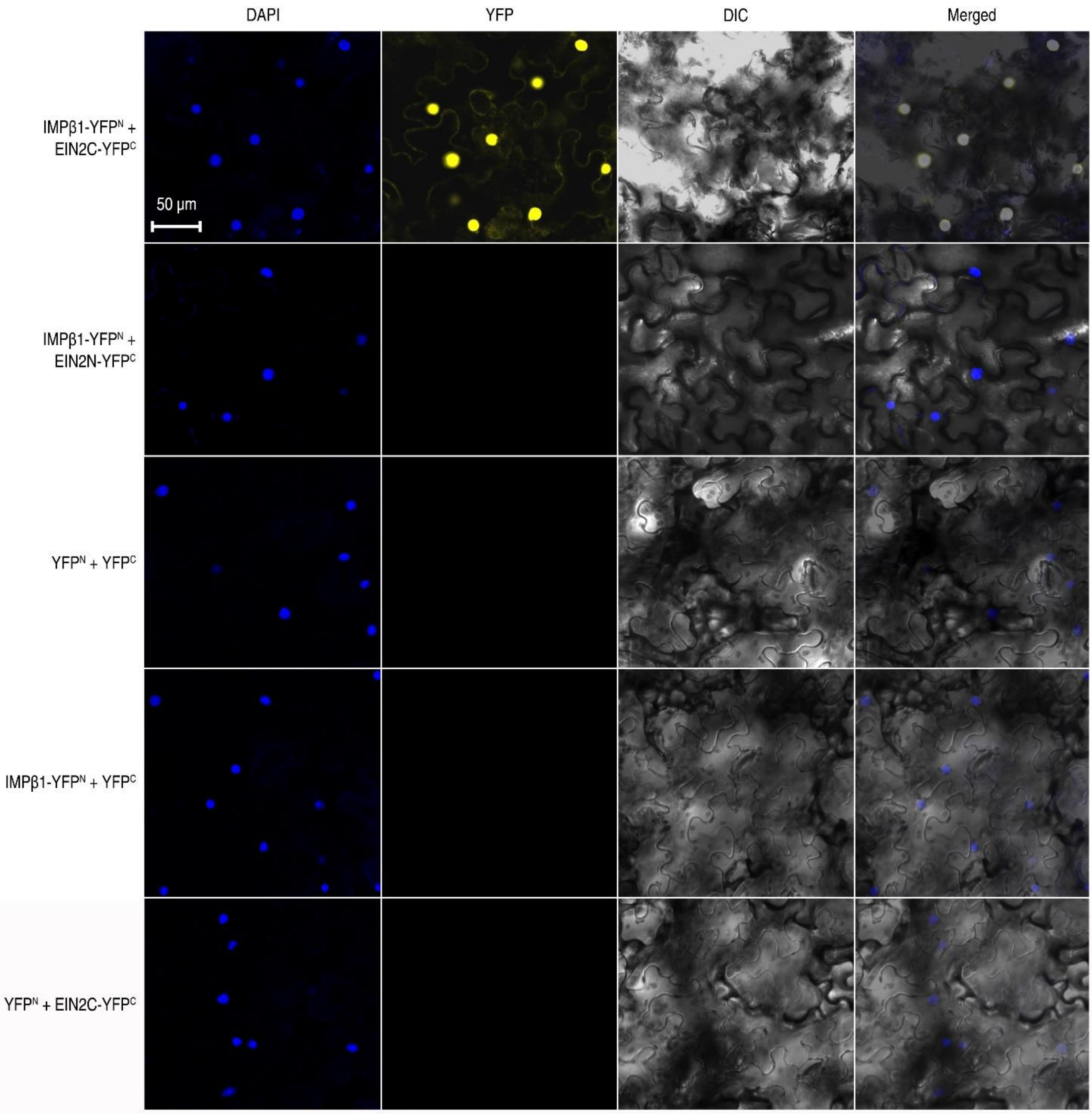


**Supplementary Figure S3.** Confocal microscopy of BiFC signals from the molecular interaction between IMPβ1-YFP$^{N}$ and EIN2C-YFP$^{C}$. The specificity in the protein-protein interaction is elucidated in contrast to multiple controls shown here and in Figure 3B. Each image represents 3 independent experiments.

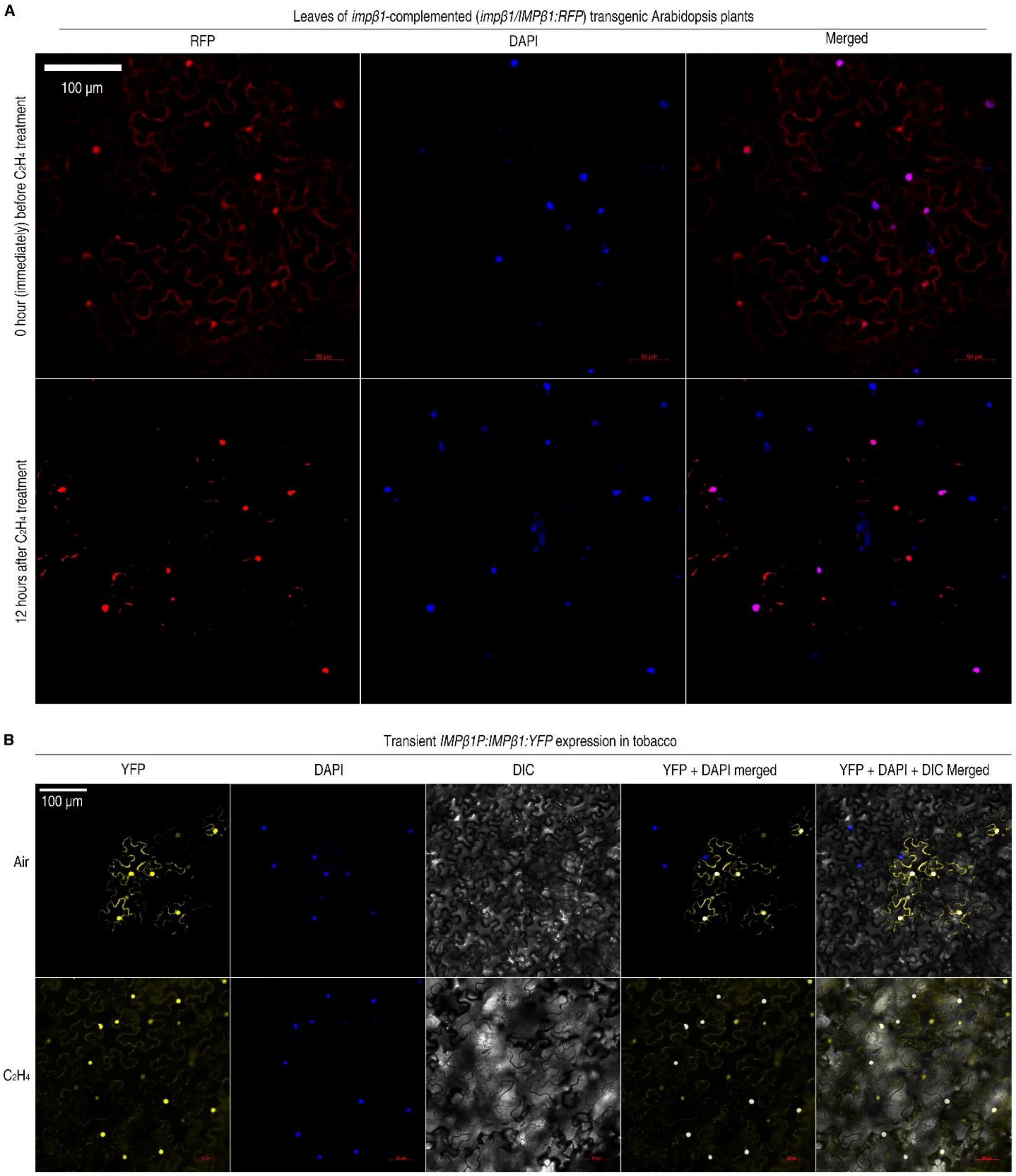


**Supplementary Figure S4.** Ethylene facilitates the localization of IMPβ1 in plant nuclei. **(A)** The IMPβ1 protein localizes to nuclei of the *impβ1*-complemented transgenic Arabidopsis plants following treatment with ethylene. **(B)** The IMPβ1-YFP fusion protein produced by transient expression in tobacco and Arabidopsis localizes to nuclei of the plants growing with ethylene. Both plants were transformed with *IMPβ1P:IMPβ1:YFP* by the leaf infiltration method and incubated in air or 10 μL/L ethylene. Three days later, the transformed leaves were excised, stained with DAPI, and observed by CLSM after additional 10 minutes. Each image represents 18 leaves from 9 plants investigated in 3 independent experiments.

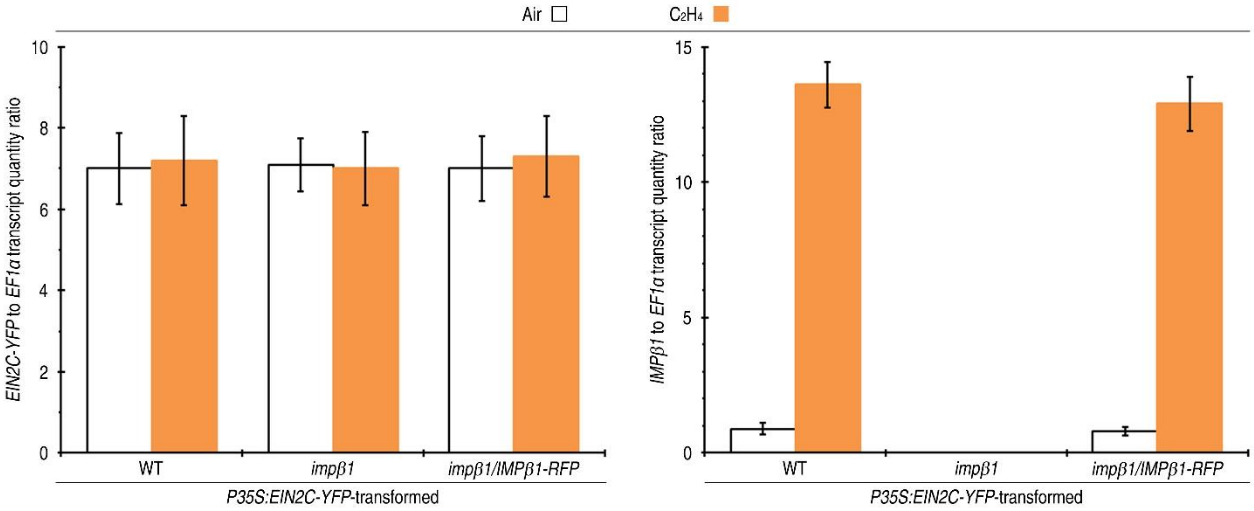


**Supplementary Figure S5.** Expression of the introduced *EIN2C-YFP* and innate *IMPβ1* genes in leaves *P35S:EIN2C:YFP*-transformed WT, *IMPβ1*, and *impβ1/IMPβ1* plants. The transformed plants were divided into two groups, remained in air in the first group and were shifted into 10 µL/L ethylene after 40 h of transformation in the second group. Additional 3 h later, RNA was isolated from the transformed leaves or equivalent leaves and analyzed by qRT-PCR. Data shown are mean values ± SDs from 6 independent experiments, each involving 15 plants tested in 3 repeats.

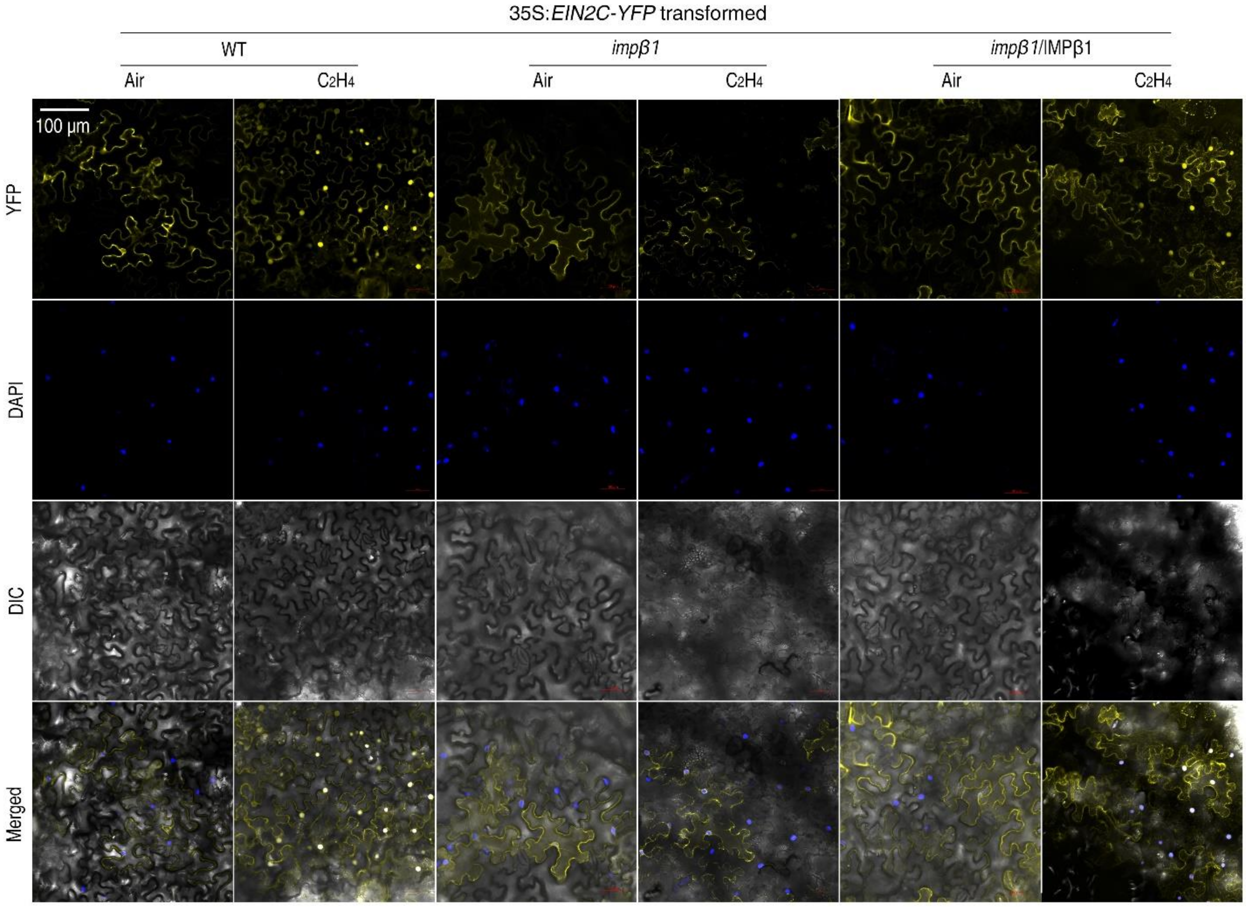


**Supplementary Figure S6.** The Nuclear import of EIN2C coincides with ethylene responses in an IMPβ1-dependent manner. The *P35S:EIN2C:YFP*-transformed WT, *IMPβ1*, and *impβ1/IMPβ1* plants. The transformed were shifted into vacuum containers that were supplied immediately with 10 μL/L ethylene. 12 h later, the other transformed leaves were excised. Excised leaves were immediately stained with DAPI and observed 10 minutes later by CLSM respectively. Each image represents 20 leaves from 20 plants tested in 3 independent experiments.

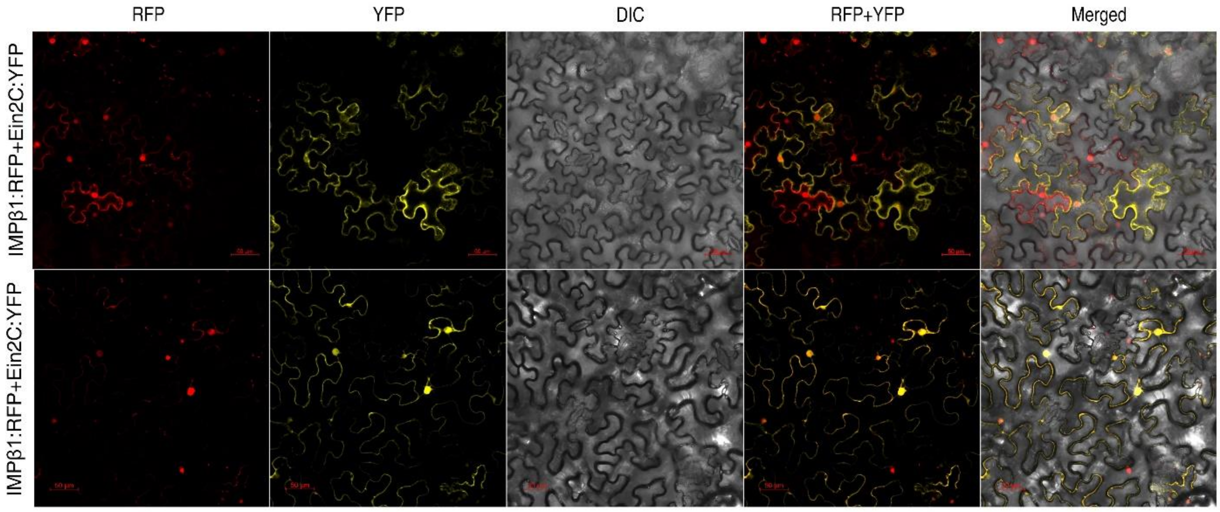


**Supplementary Figure S7.** IMPβ1 and EIN2C are co-localize to nuclei in response to ethylene. Leaves of 30-day-old tobacco plants were co-transfected with the 35S:*IMPβ1-RFP* and 35S:*EIN2C-YFP* constructs. 48 h later, transfected plants were shifted into vacuum containers. The containers were supplied immediately with ethylene in the treatment group but not in the control group. These leaves were observed under a laser confocal microscope. Each of the images shown here represents 20 leaves from 10 plants tested in 3 independent experiments.
